# Dynamic transcriptome profiles within spermatogonial and spermatocyte populations during postnatal testis maturation revealed by single-cell sequencing

**DOI:** 10.1371/journal.pgen.1007810

**Published:** 2019-03-20

**Authors:** Kathryn J. Grive, Yang Hu, Eileen Shu, Andrew Grimson, Olivier Elemento, Jennifer K. Grenier, Paula E. Cohen

**Affiliations:** 1 Center for Reproductive Genomics, Cornell University, Ithaca, NY, United States of America; 2 Department of Biomedical Sciences, Cornell University, Ithaca, NY, United States of America; 3 Englander Institute for Precision Medicine, Weill Cornell Medicine, New York, NY, United States of America; 4 Department of Molecular Biology and Genetics, Cornell University, Ithaca, NY, United States of America; The University of North Carolina at Chapel Hill, UNITED STATES

## Abstract

Spermatogenesis is the process by which male gametes are formed from a self-renewing population of spermatogonial stem cells (SSCs) residing in the testis. SSCs represent less than 1% of the total testicular cell population in adults, but must achieve a stable balance between self-renewal and differentiation. Once differentiation has occurred, the newly formed and highly proliferative spermatogonia must then enter the meiotic program in which DNA content is doubled, then halved twice to create haploid gametes. While much is known about the critical cellular processes that take place during the specialized cell division that is meiosis, much less is known about how the spermatocytes in the “first-wave” in juveniles compare to those that contribute to long-term, “steady-state” spermatogenesis in adults. Given the strictly-defined developmental process of spermatogenesis, this study explored the transcriptional profiles of developmental cell stages during testis maturation. Using a combination of comprehensive germ cell sampling with high-resolution, single-cell-mRNA-sequencing, we have generated a reference dataset of germ cell gene expression. We show that discrete developmental stages of spermatogenesis possess significant differences in the transcriptional profiles from neonates compared to juveniles and adults. Importantly, these gene expression dynamics are also reflected at the protein level in their respective cell types. We also show differential utilization of many biological pathways with age in both spermatogonia and spermatocytes, demonstrating significantly different underlying gene regulatory programs in these cell types over the course of testis development and spermatogenic waves. This dataset represents the first unbiased sampling of spermatogonia and spermatocytes during testis maturation, at high-resolution, single-cell depth. Not only does this analysis reveal previously unknown transcriptional dynamics of a highly transitional cell population, it has also begun to reveal critical differences in biological pathway utilization in developing spermatogonia and spermatocytes, including response to DNA damage and double-strand breaks.

## Author summary

Spermatogenesis is the process by which male gametes–mature spermatozoa–are produced in the testis. This process requires exquisite control over many developmental transitions, including the self-renewal of the germline stem cell population, commitment to meiosis, and ultimately, spermiogenesis. While much is known about molecular mechanisms regulating single transitions at single time points in the mouse, much less is understood about how the spermatogenic progenitor cells, spermatogonia, or the meiotic cells, spermatocytes, change during testis maturation. Our single-cell-mRNA-sequencing analysis is the first to profile both spermatogonia and spermatocytes from neonatal mice through adulthood, revealing novel gene expression dynamics and differential utilization of biological pathways. These discoveries help us to understand how the spermatogenic progenitors of this population modulate their activity to adapt to a changing testicular environment. Furthermore, they also begin to explain previously-observed differences—and deficiencies—in spermatocytes that are derived from the “first wave” of spermatogenesis in juvenile mice. Overall, this dataset is the first of its kind to comprehensively profile gene expression dynamics in male germ cell populations during testis development, enriching our understanding of the complex and highly-orchestrated process of spermatogenesis.

## Introduction

Mammalian spermatogenesis requires proper establishment of the spermatogonial stem cell (SSC) pool, which resides within the seminiferous tubules of the testis and supports life-long germ cell development [1]. These progenitors give rise to all the differentiating germ cells, ranging from spermatogonia to spermatocytes to spermatids, and finally to mature spermatozoa. Despite the essential nature of this process, the genetic regulatory mechanisms underlying the many complex cellular transitions, and the maturation of this system during testis development, have yet to be fully described.

Gamete development in the mouse relies on a rare population of primordial germ cells, the bi-potential progenitors of all gametes, which are specified at embryonic day (E) 6.25 [2]. These cells migrate to and colonize the developing gonad, arriving at the genital ridge from E10.5 [3], and undergo abundant proliferation until E13.5. At this time, germ cells developing in an XX (female) gonad will enter the meiotic program as “oocytes”, while germ cells developing in an XY (male) gonad will become “prospermatogonia”, remaining relatively non-proliferative until shortly after birth [4,5]. Prospermatogonia are then able to adopt several fates [6]: in early postnatal life, a subset of these cells differentiate immediately into spermatogonia and continue to progress through spermatogenesis, to constitute the “first wave” of spermatogenesis. A second subset of prospermatogonia will undergo apoptosis, while the remaining prospermatogonia will become established within the testicular stem cell niche soon after birth, to become the self-renewing SSC population which will support “steady-state” spermatogenesis throughout life. This germline stem cell population makes up less than 1% of the cells of adult testes [7], and must balance self-renewal and differentiation to maintain a healthy male gamete supply. Thus, the first cohort of meiotically-active male germ cells enters the meiotic program without first entering a self-renewal SSC phase, clearly differentiating the first wave of spermatogenesis from the other subsequent waves.

SSCs are triggered to enter spermatogenesis coincident with a burst of retinoic acid (RA), which induces both the spermatogonial divisions and the entry into prophase I of meiosis [8–11]. Thus, in mice, male meiotic entry commences around postnatal day (PND) 10, in response to RA-induced expression of key genes, including ‘Stimulated by Retinoic Acid 8’ (*Stra8*) [8–11]. Spermatocytes execute many essential meiotic events including creation of double-strand breaks, synapsis of homologous chromosomes, and DNA repair and crossover formation, all of which are critical to proper segregation of homologs in the first meiotic division. Failure to properly execute any of these steps is known to result in potential chromosome mis-segregation, non-disjunction events, aneuploidy, and infertility (reviewed comprehensively in Baarends *et al* [12] and Gray & Cohen [13]).

While the developmental transitions which underlie germ cell differentiation and maturation have been broadly defined, the gene regulatory underpinnings of these transitions remain largely uncharacterized. Concurrent with our work presented herein, many groups have also investigated developmental transitions within the testis using single-cell sequencing, and have begun to shed some light upon genetic regulatory mechanisms of these processes [14–18]. Intriguingly, several new cell types have been identified, including previously unidentified somatic cells [14], and murine spermatogenesis has been extensively compared to human spermatogenesis [15], emphasizing the translational impact of these types of studies. A caveat of these studies, however, is their focus on single time points, or utilization of cell enrichment protocols that may bias the output. In this manuscript, we have performed the first single-cell sequencing developmental time series of the male mouse germline with comprehensive sampling, thereby capturing all germ cell types through the progression of postnatal testis maturation. The advent of single cell transcriptomics provides an invaluable tool for understanding gene expression dynamics at very high resolution in a large number of individual cells in parallel. Furthermore, single-cell sequencing reveals heterogeneity and potential plasticity within cell populations, which bulk mRNA sequencing is unable to accomplish, making it an ideal tool for profiling germ cell populations which rapidly progress through myriad developmental transitions.

We demonstrate that germ cells display novel gene regulatory signatures during testis development, while cells positive for single protein markers have the capacity to change dramatically with age, and therefore cells of a particular “identity” may differ significantly from postnatal to adult life. Intriguingly, we have also begun to identify differential expression of genes in critical biological pathways which may contribute to observed differences in the first-wave of spermatogenesis [19,20]. Dissecting the complex dynamics of these developmental transitions can provide critical information about the transcriptional landscape of both SSCs, spermatogonia, and spermatocytes, and the regulatory mechanisms that underlie the formation of a dynamic and functional complement of germ cells to support life-long spermatogenesis.

## Results

### Single-cell sequencing from testes of different developmental ages robustly defines germ cell populations

**Fig 1:**
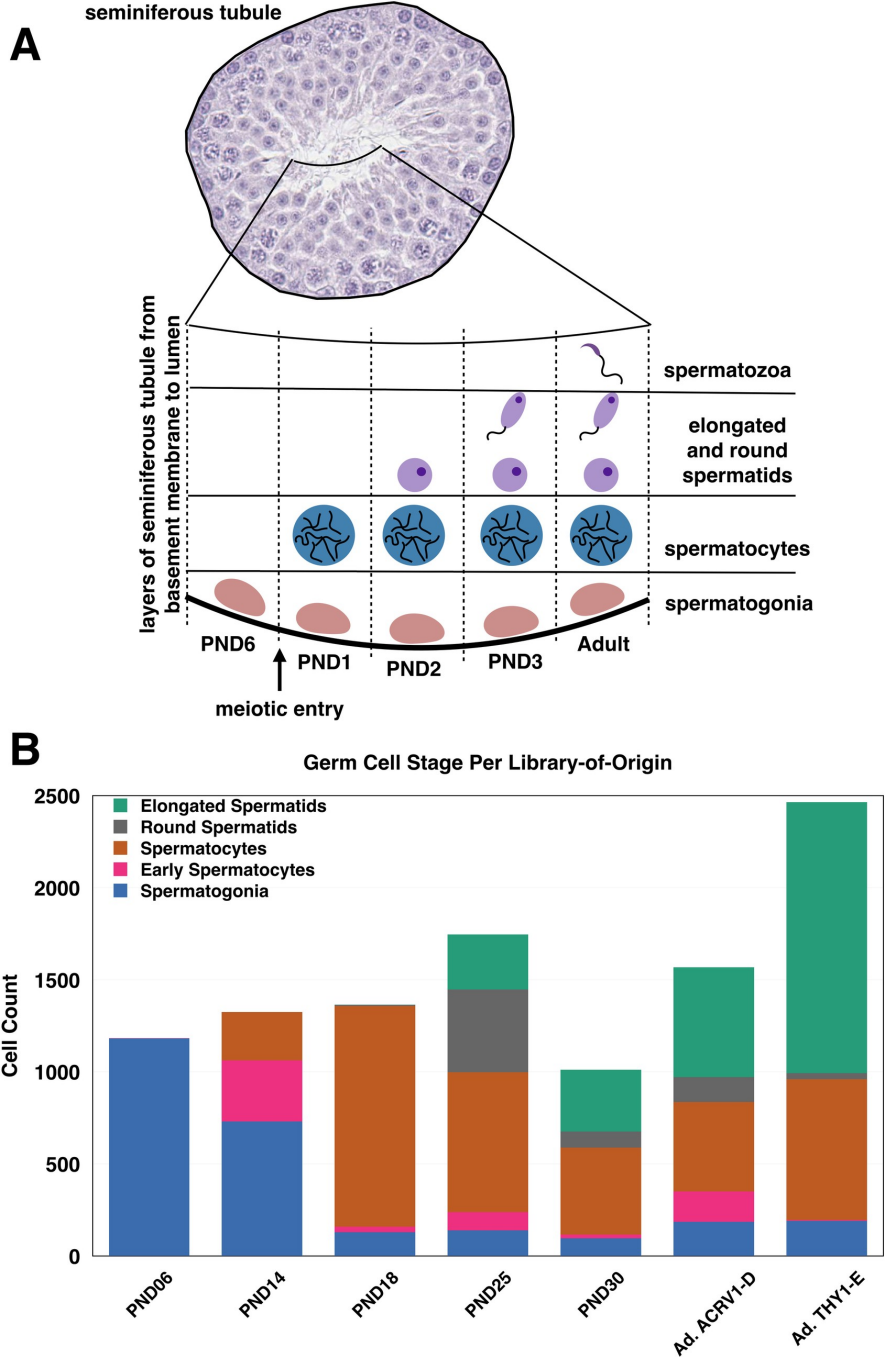
Germ cells profiled in single-cell sequencing analysis are representative of known biology of the developing testis. A) Schematic of the developing testis with germ cell representation at each time point. Only spermatogonia are present during the first week of life, until meiotic entry at PND10, after which germ cells can commit meiosis and progress through spermatogenesis and spermiogenesis, producing the first mature spermatozoa from the first wave around PND30. B) Germ cell composition by proportion and absolute cell number from each library-of-origin. “Ad” indicates adult libraries, ACRV1-D indicates ACRV1+ depletion. and THY1-E indicates THY1+ enrichment.

**Fig 2:**
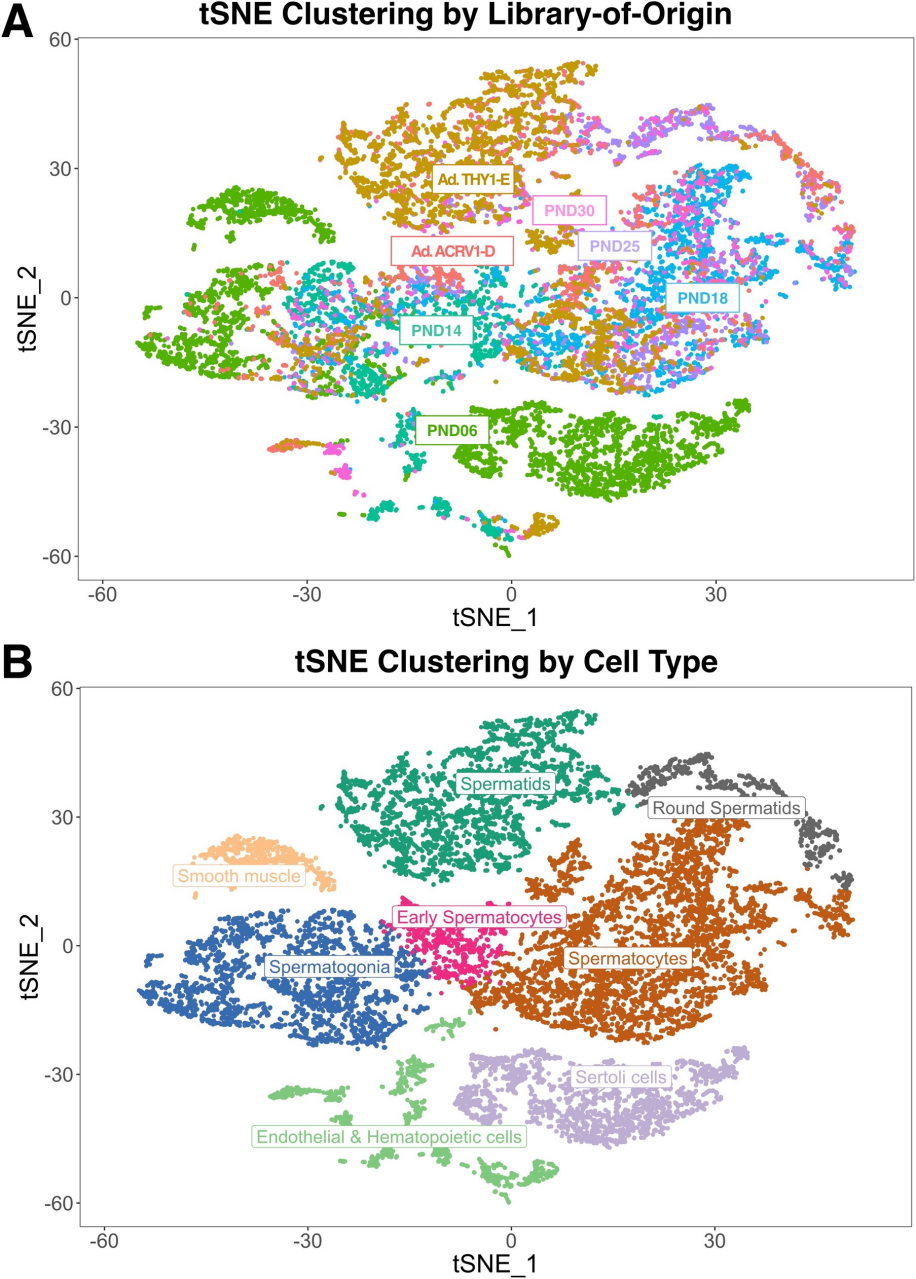
Clustering of single-cell data into libraries-of-origin and cell type classifications. A) tSNE representation of all cells with >500 detected genes and >2,000 UMI (unique molecular identifier) counts, color-coded by library-of-origin. “Ad” indicates adult libraries, ACRV1-D indicates ACRV1+ depletion, and THY1-E indicates THY1+ enrichment. B) tSNE representation of all cells with >500 detected genes and >2000 UMIs, color-coded by cell type classification.

Mouse testes were collected at several postnatal time points, selected to represent distinct stages of germline development: postnatal day (PND) 6 (during SSC specification), PND14 (first appearance of pachytene spermatocytes during the first wave), PND18 (pachytene and diplotene spermatocytes from the first wave present), PND25 (spermatids present) and PND30 and adult (spermatozoa present) (**Fig 1A**) and subjected to single-cell RNAseq. The tissue was dissociated, and the resulting slurry subjected to 30% Percoll sedimentation to remove debris. The PND18 cell suspension was split and processed both with and without Percoll sedimentation as a technical control; due to similarities between libraries, the data from these libraries was thereafter combined (**S1 Fig**). Additionally, due to the proportionally high representation of sperm in the adult testis, it was necessary to increase representation of other germ cell types from these samples. To accomplish this goal, an adult testis suspension post-Percoll sedimentation was split in half and either positively magnetically-cell-sorted (MACS) for the cell surface marker THY1, in an attempt to enrich for spermatogonia [21], or negatively MACS-sorted for ACRV1, in an attempt to deplete testicular sperm [22]. While neither strategy can accomplish complete enrichment of spermatogonia or removal of spermatozoa, respectively, both adult libraries had a representative sample of all germ cell types (**Fig 1B**), and are therefore treated as adult replicates in these data. For each single-cell testis suspension, 4–5,000 cells per mouse were processed through the 10X Genomics Chromium System using standard protocols for single cell RNA sequencing. Libraries were sequenced to an average depth of 98M reads; on average, 91% of reads mapped to the reference genome. After standard data processing, we obtained gene expression profiles for approximately 1,100–2,500 cells per library (**Figs 1B and 2A, S1 Table**) with representation of between 2,500–5,000 genes per cell, and an average of nearly 32,000 mapped reads/cell post-normalization (also known as “UMI”; unique molecular identifier counts) (**S2 Fig**), all of which indicate the robustness of the sequencing method and comparability to UMI counts in other similar studies [14,15,17].

**Fig 3:**
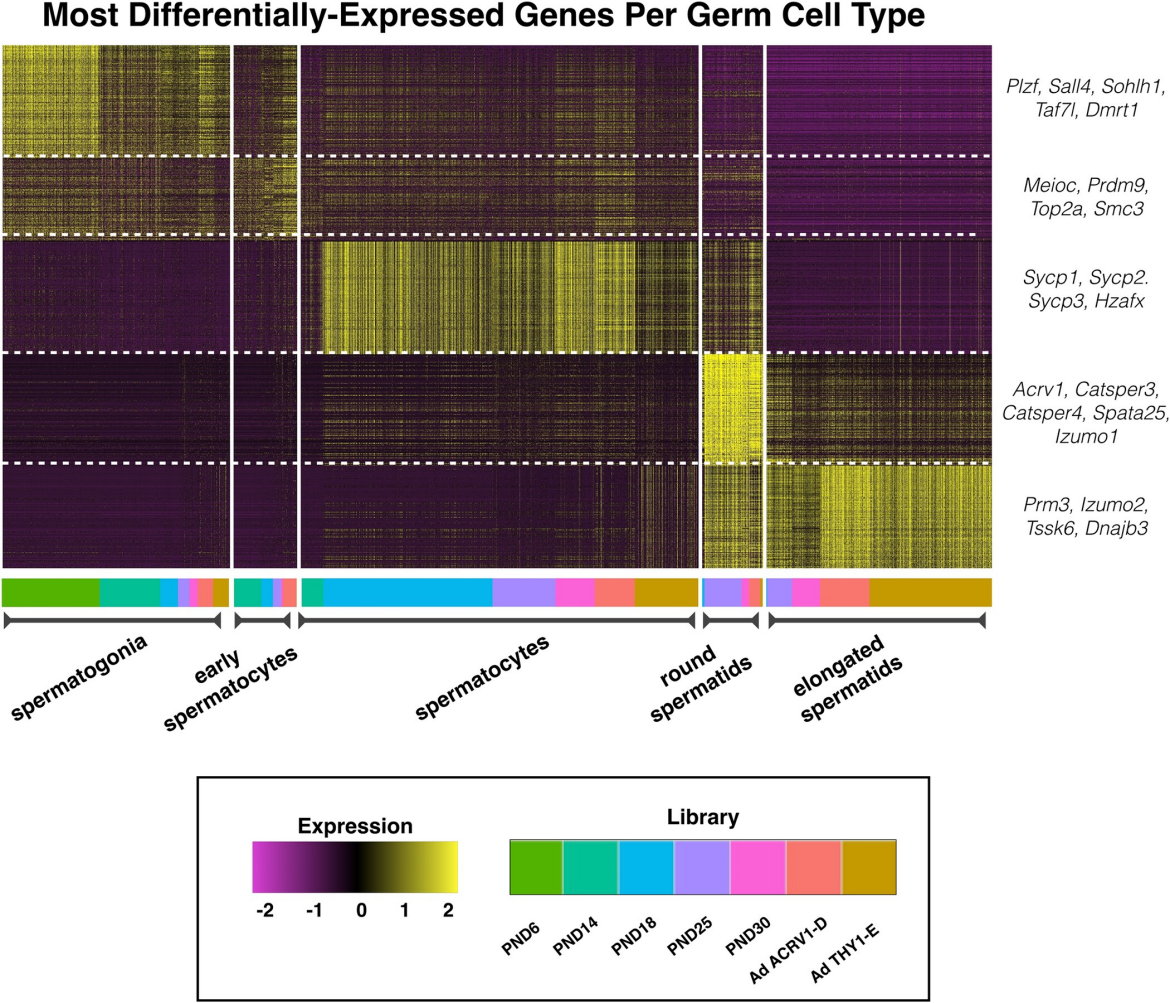
Marker gene heatmap of all germ cell types reveals characteristic signatures. Heatmap of most-differentially-expressed marker genes per germ cell type. Color bar at the bottom indicates library-of-origin time point for cells within each block. Expression is represented as a z-score ranging from -2 to 2. Notable marker genes for each germ cell type are highlighted to the right of the heatmap. “Ad” indicates adult libraries, ACRV1-D indicates ACRV1+ depletion, and THY1-E indicates THY1+ enrichment.

Primary cell clusters revealed by Seurat (**S3 Fig**) were identified and merged into superclusters (**Fig 2B**) based on characteristic marker gene expression (**S2 Table**). While somatic cells were evident in the clustering analysis, including Sertoli cells, smooth muscle, and epithelial and hematopoetic cells, they represent a minority (fewer than 25%) of the total cells profiled (**S1 Table**). In particular, Sertoli cells were primarily derived from the PND6 library (**Fig 2**) and exhibit expression of known immature-, mature-, and pan-Sertoli cell markers [23–28] (**S4A Fig**). This biased recovery is likely due to their increased representation at the neonatal time point, as well as processing steps which effectively removed these cells, which become considerably larger in older mice. This reduced retention of somatic cells in the processing of testes from older mice is consistent with other single-cell sequencing studies of the testis, and can only be mitigated by different dissociation or filtering methods and/or cell sorting [14]. Therefore, somatic cells were excluded from gene expression analysis during testis maturation, which focused on the spermatogonial and spermatocyte populations. Analysis of differentially expressed genes between cell clusters identified characteristic marker genes in each cell type, including *Zbtb16* (*Plzf*), *Sall4*, *Sohlh1*, and *Dmrt1* in spermatogonia; *Meioc*, *Prdm3*, *Top2a*, and *Smc3* in early spermatocytes; *Sycp1/2/3* and *H2afx* in spermatocytes; *Acrv1*, *Izumo1*, and *Catsper3/4* in round spermatids; and *Prm3*, *Izumo3*, and *Tssk6* in elongated spermatids (**Figs 3, S5 & S6, S2 Table**).

Critically, the germ cell type classifications are representative of the known timeline of the developing testis (**Fig 1B**), with only spermatogonia present at PND6, some early spermatocytes present at PND14, much greater representation of those spermatocytes at PND18, and appearance of more differentiated round and elongated spermatids from PND25 onwards. Interestingly, we observed the greatest enrichment of spermatids in the positively THY1-sorted adult sample, likely due to non-specific binding of the antibody to the developing acrosome. Despite this, the library contained strong representation of spermatogonia and spermatocytes and was therefore retained in the analysis. The negative ACRV1 sorting for the other adult sample retained representation of all germ cell types in the adult testis, including spermatogonia, which would otherwise have been poorly represented due to the much greater abundance of more differentiated cell types. Overall, both adult samples provide excellent representation for all germ cell types present in the adult testis and are therefore included in this sampling analysis.

Importantly, we also observe the expected down-regulation of X-linked genes during meiosis I. Numerous studies have demonstrated that Prophase I progression is associated with only partial synapsis of the X and Y chromosomes at the pseudo-autosomal region [29–31]. As a result, the sex chromosomes undergo progressive silencing throughout the unsynapsed chromatin, with complete silencing by pachynema [31–34]. As a positive validation of appropriate spermatocyte classification, a random sampling of X-linked genes were analyzed for their expression across cell types (**S4B Fig**). Despite several patterns of X-linked gene expression among cell clusters, the spermatocyte supercluster exhibits very little to no detection of all profiled X-linked genes, again confirming the robustness of our cell-type classification methods.

Cell-free RNA contamination from lysed cells is a well-known confounding feature in single-cell sequencing libraries, as highly-expressed transcripts from even a small number of lysed cells can become incorporated in the gel bead emulsions of single-cell microfluidics devices [35]. As a result of the incorporation of these transcripts into libraries of cells from which they did not originate, cells which do not endogenously express such transcripts can appear to have low levels of expression of these markers, potentially confounding data analysis due to technical artefacts. In this data set, genes highly expressed in elongated spermatids/sperm were detected at low levels in other cell types, including somatic cells, nearly exclusively in libraries derived from testes aged 25 days or older (**S7 and S8 Figs**), the only samples in which spermatids are present. Therefore, we believe the detection of these transcripts in both spermatogonia and spermatocytes of older mice is due to contamination from cell-free RNA derived from lysed spermatids. To mitigate the age-related biases this signal might pose in down-stream analysis, markers of the spermatid/sperm population (genes with a greater than 20:1 ratio of expression between spermatids and other germ cell types), such as *Prm1/2*, were removed from further analysis (**S3 Table**).

### Spermatogonia display characteristic transcriptional signatures, but also novel gene expression dynamics, during testis maturation

**Fig 4:**
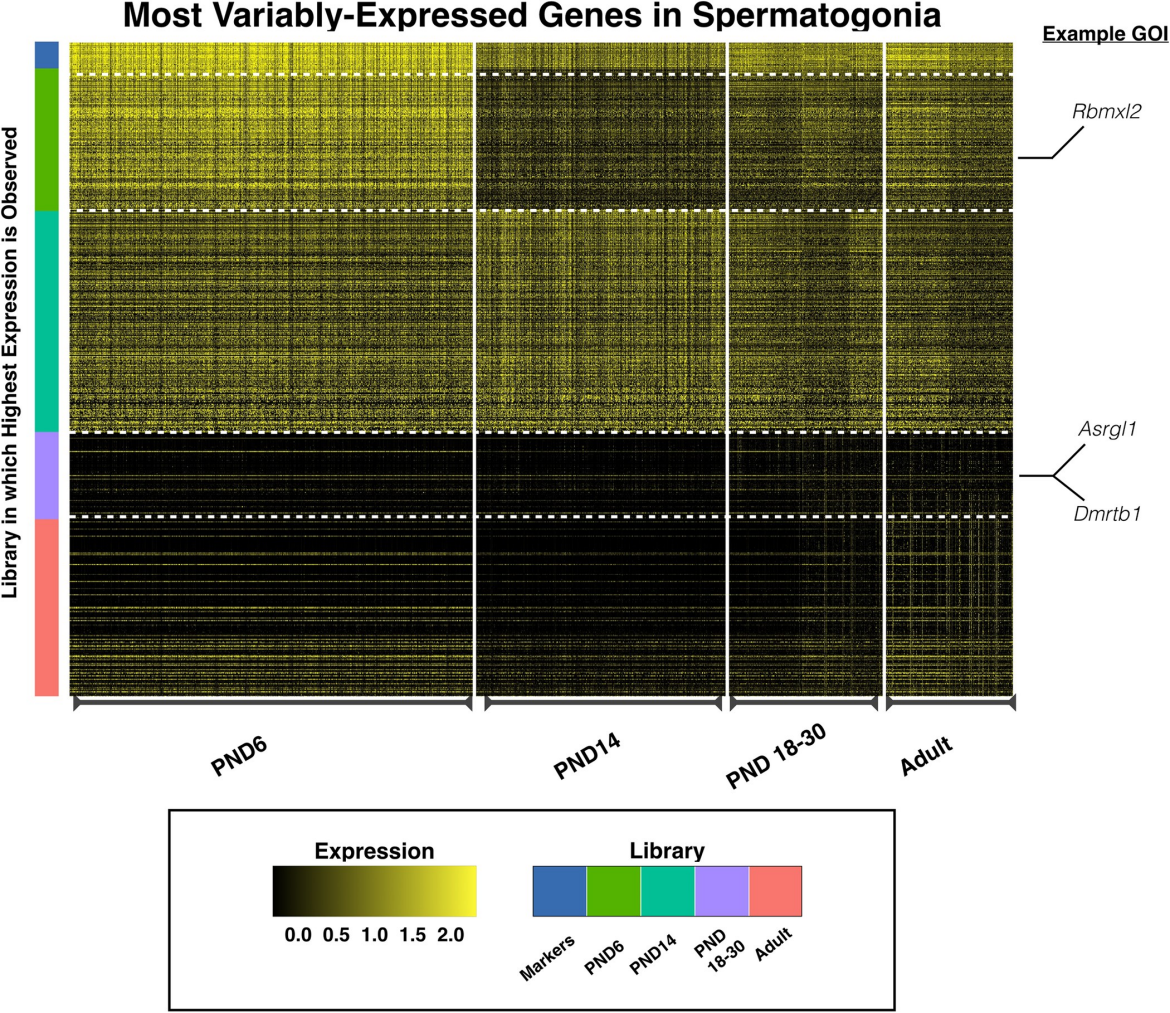
Analysis of variably-expressed genes in spermatogonia. MAST analysis was used to determine genes variably expressed with age specifically in spermatogonia, represented in a heatmap. All genes represented in the heatmap and listed in **S4 Table** are differentially expressed with the exception of the marker genes [at the top] which remain consistently expressed. PND18-30 time points have been merged to increase representation of this rare cell type at those time points. Similarly, adult time points have also been merged. Individual cells are plotted along the x-axis and the library of origin is indicated at the bottom of the heatmap. Individual genes are plotted on the y-axis and the color bar at the left indicates library-of-origin from which highest expression is observed. Expression is scaled, ranging from 0 to 2.5.

To better understand the developmental transitions that spermatogonia undergo as mice age, genes variably-expressed with age were identified by Model-based Analysis of Single Cell Transcriptomics (MAST) [36] (**S4 Table**) and visualized as a heatmap (**Fig 4**). As spermatogonia become proportionally rarer with age (and therefore later-aged individual libraries experience lower representation), spermatogonia from libraries PND18, PND25, and PND30 were merged with each other, as were spermatogonia from the two adult samples. While marker genes, indicated in the top row, remain quite constant over time, clear and novel differences can be observed in spermatogonial gene expression over time, particularly in spermatogonia derived from PND6 testes. Several genes, including those noted to the side of the heatmap, are observed to have robust differential expression over time, and may play a role in the establishment and growth of this spermatogonial pool which will support lifelong spermatogenesis.

**Fig 5:**
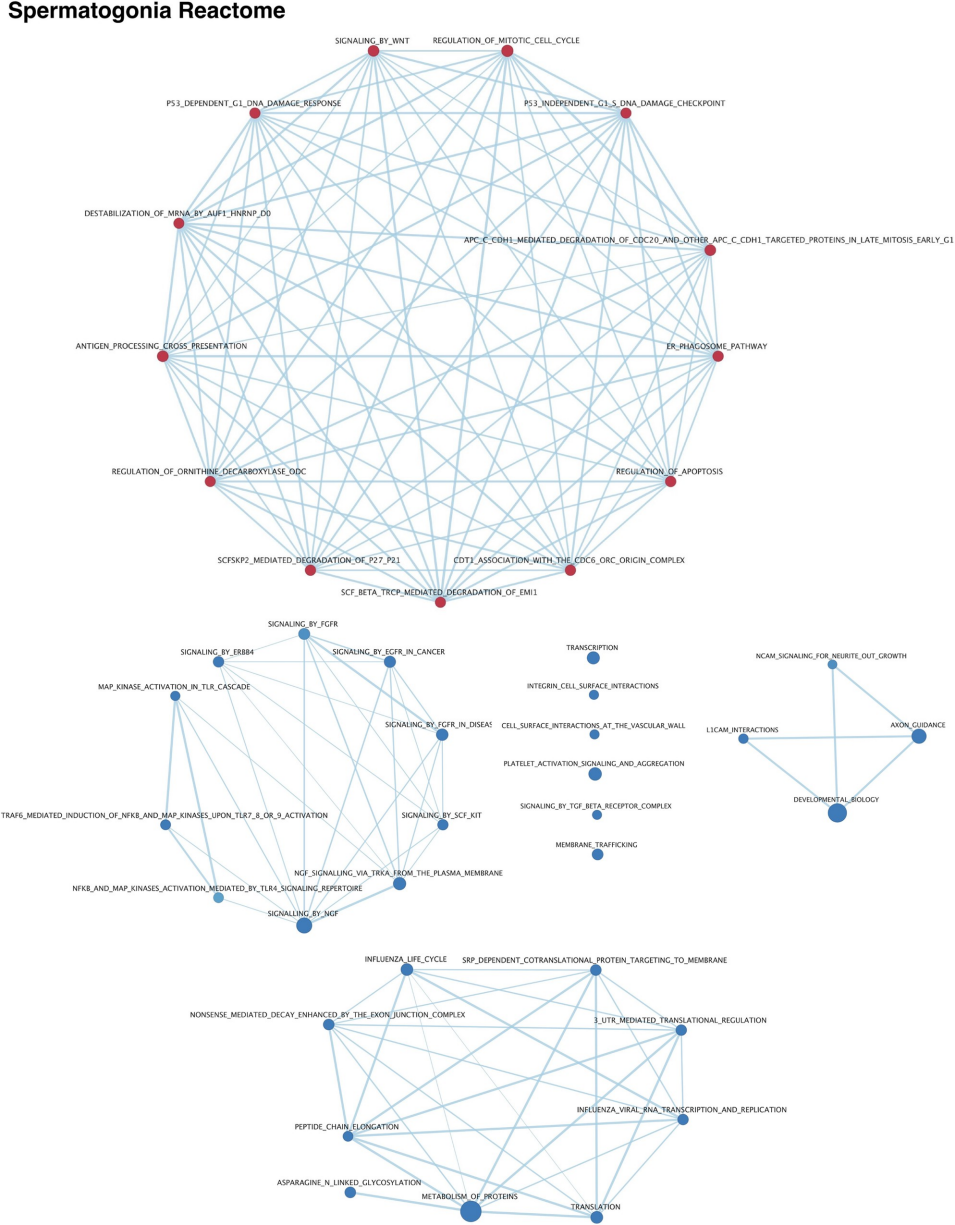
Differential Reactome pathway utilization in spermatogonia with age. Gene set enrichment analysis of variably-expressed genes in the Reactome database was visualized in Cytoscape. Results were filtered on a false discovery rate <0.05, and a gene set list >45 genes. Red nodes indicate pathways upregulated with testis age while blue nodes indicate pathways down-regulated with testis age. Edges indicate connections and overlap between pathways.

In addition to MAST analysis for variable expression, Gene Set Enrichment Analysis (GSEA) Time Series analysis [37] of Reactome pathways revealed differentially-utilized pathways, which were then visualized using Enrichment Map in Cytoscape [38,39]. GSEA of variably-expressed genes in spermatogonia from mice of different ages reveals significant changes in many pathways, including increasing expression of genes related to RNA destabilization and protein degradation as well as WNT signaling, and decreasing expression of genes related to asparagine metabolism, various signaling pathways including TGFB, FGFR, and KIT, and transcriptional regulation (**Figs 5 & S9A, S5 Table**). In particular, many critical signaling receptors and ligands, including *Kit* and *KitL* [40–43], as well as *Fgf8* and *Fgfr1* [44,45], exhibit downregulation in spermatogonia derived from mice of increasing age, consistent with potentially altered paracrine signaling around the basement membrane of the seminiferous tubules during testis maturation.

### Spermatocytes from the first wave of spermatogenesis are transcriptionally distinct from steady-state spermatocytes

**Fig 6:**
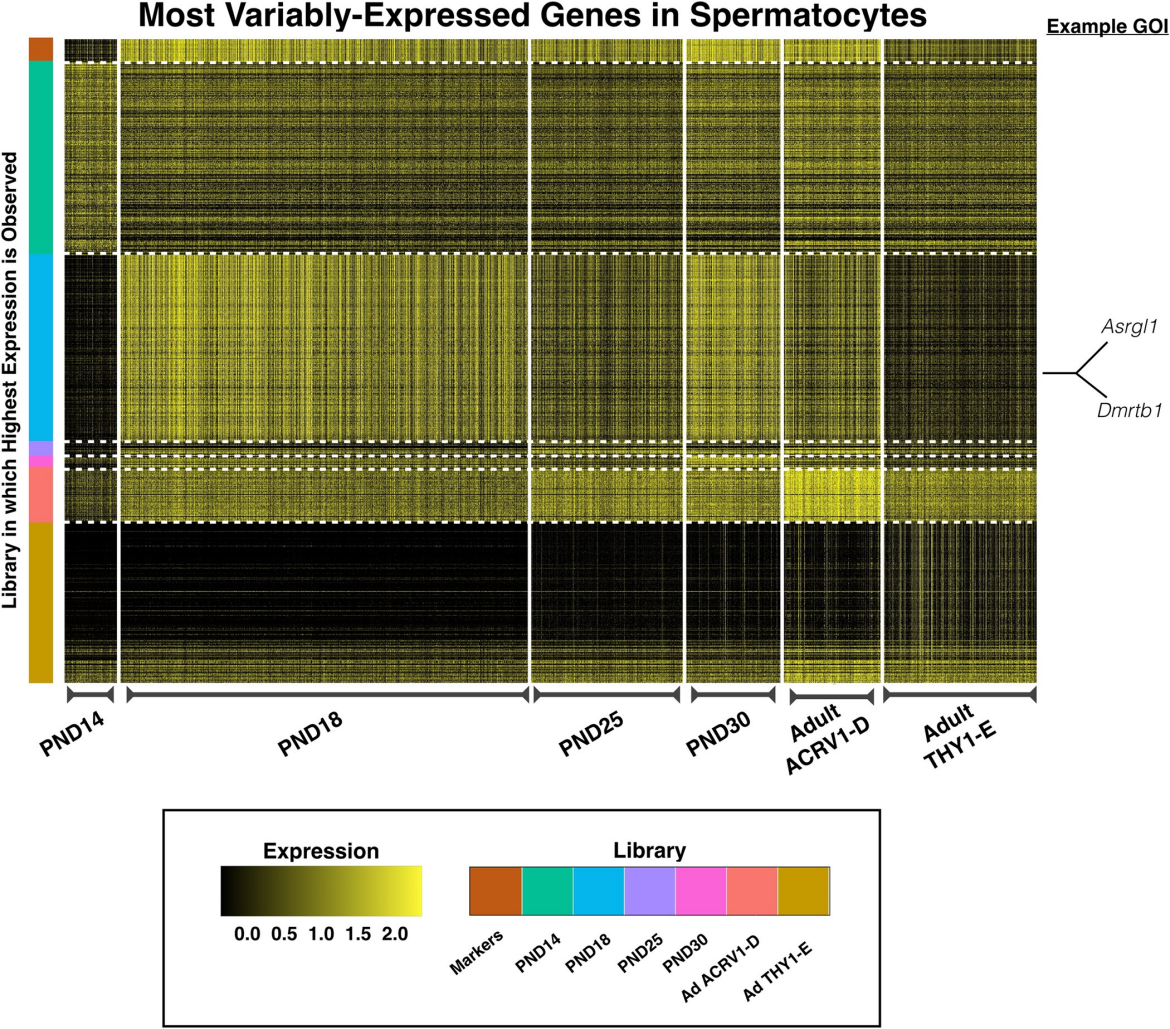
Analysis of variably-expressed genes in spermatocytes. MAST analysis was used to determine genes which are variably expressed with age specifically in spermatocytes, represented in a heatmap. All genes represented in the heatmap and listed in **S6 Table** are differentially expressed with the exception of the marker genes [at the top] which remain consistently expressed. Individual cells are plotted along the x-axis and the library of origin is indicated at the bottom of the heatmap. Individual genes are plotted on the y-axis and the color bar at the left indicates library-of-origin from which highest expression is observed. Expression is scaled, ranging from 0 to 2.5. “Ad” indicates adult libraries, with ACRV1-D indicating ACRV1+ depletion, while THY1-E indicates THY1+ enrichment.

It has been well established that meiotic regulation is distinct in the first wave of meiosis compared to that in subsequent waves [6,19,20]. Thus, we sought to characterize this phenomenon in terms of the transcriptome profile of spermatocytes at discrete developmental time points. Spermatocytes from the first meiotic wave compared to steady-state (adult) ages were also subjected to MAST analysis, as described above (**Fig 6**, **S6 Table**). For this analysis, spermatocytes were abundant enough from all libraries that each time point could be considered separately, except for PND6 in which spermatocytes are not yet present. Notably, spermatocytes from PND14, which are only just beginning Prophase I, demonstrate very distinct gene expression patterns from spermatocytes at later time points and are not representative of the full spectrum of meiotic cell types, as expected. Some genes, including those noted to the side of the heatmap, show robust differential expression with age, highlighting differences between spermatocytes derived from the first-wave (PND18) in contrast those which are derived from a self-renewing SSC population (adult). Therefore, these genes were chosen for further analysis and orthogonal validation.

**Fig 7:**
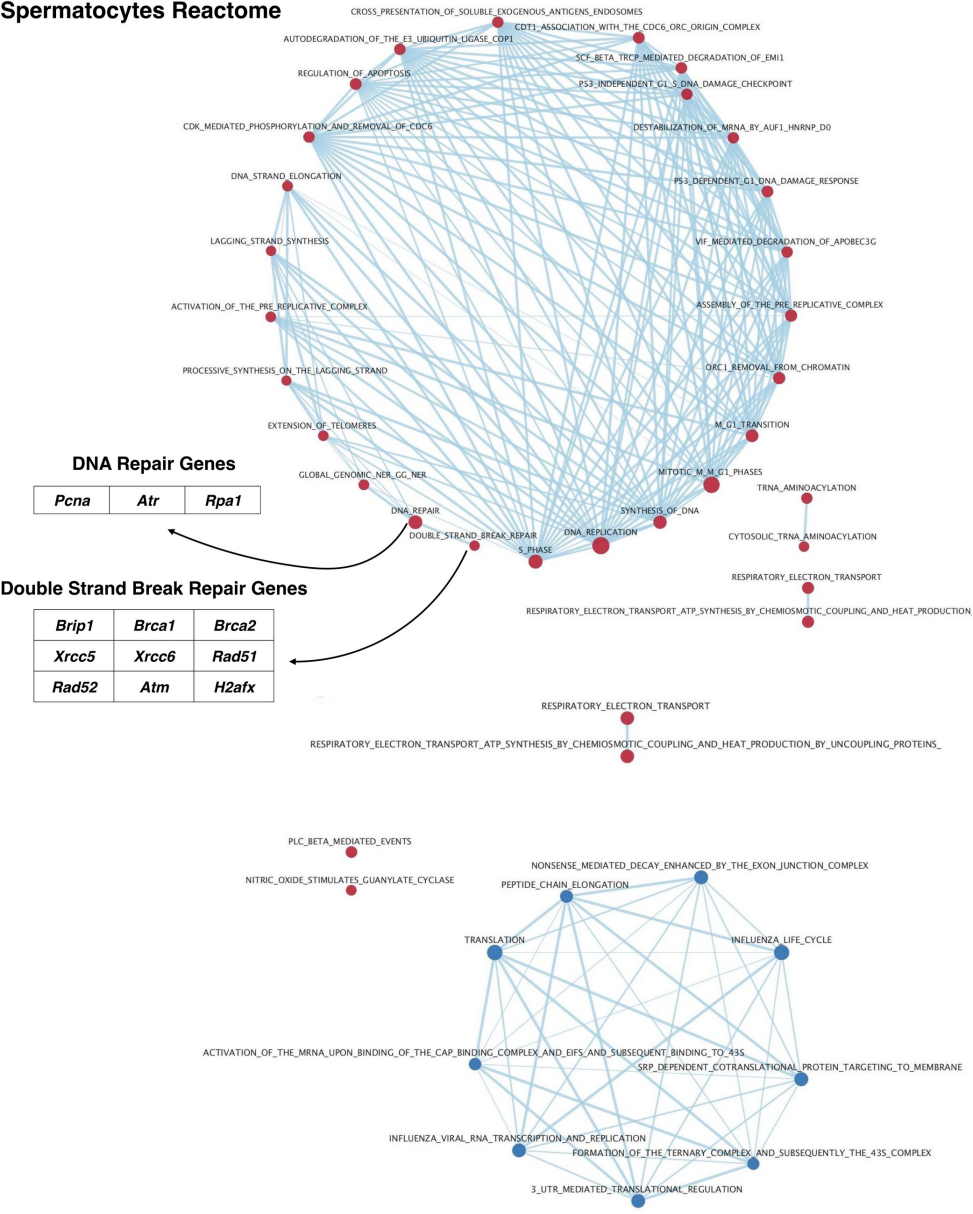
Differential Reactome pathway utilization in spermatocytes with age. Gene set enrichment analysis of variably-expressed genes in the Reactome database was visualized in Cytoscape. Results were filtered on a false discovery rate <0.05, and a gene set list >15 genes. Red nodes indicate pathways upregulated with time while blue nodes indicate pathways down-regulated with time. Edges indicate connections and overlap between pathways. The tables to the left of the diagrams identify notable genes represented in these pathways.

GSEA time series analysis of Reactome pathway enrichment of variably expressed genes in spermatocytes also reveals intriguing differentially utilized pathways. From this analysis, we observe decreasing expression of genes related to translation and post-transcriptional regulation, and increasing expression of genes related to DNA replication, double strand break repair, and cell cycle regulation (**Figs 7 & S9B, S7 Table**). Most notable in the list of genes upregulated in spermatocytes of increasing age are those known to be essential to DNA repair, meiotic progression, and crossover formation including *Brip1* [46], *Brca1* and *Brca2* [47–49], *Rad51* [50], *H2afx* [51] and *Atm* [52]. Many of these pathways, particularly those related to double strand break repair (which initiates meiotic recombination), may be crucial for understanding the molecular mechanisms underlying fundamental differences in first-wave spermatocytes.

### Validation of differentially expressed genes demonstrates dynamic protein expression within a defined cell type during testis maturation

Candidate genes of interest (GOIs) identified in the single-cell sequencing data were investigated using immunofluorescence, which allowed us to validate changes in protein expression in the context of the native testis tissue. We used paraffin-embedded testis tissue sections at postnatal ages PND7, PND13, PND22, and 8 weeks of age to characterize the same range of postnatal testis development as was captured in the single-cell RNAseq data set. In an effort to reduce batch effects emerging from testis isolation on different days, and in light of the subtlety of some of the differential protein expression, we prioritized simultaneous processing over exact age-matching.

GOIs were selected by meeting several criteria including: representation across several biological pathways, significant differential expression in a given cell type between mice of different ages, and availability of a commercial immunofluorescence-verified and mouse-reactive antibody. For all validation of spermatogonial candidates, double-staining was performed with an antibody against ‘Promyelocytic Leukemia Zinc Finger Protein’ (PLZF; aka ZBTB16), a well-characterized marker of undifferentiated spermatogonia [53–55]. For all validation of spermatocyte candidates, double-staining was performed with an antibody against ‘Synaptonemal Complex Protein 3’ (SYCP3), to allow for visualization and staging of Prophase I-staged cells [13,56]. SYCP3 marks the nuclei of spermatocytes through leptotene, zygotene, pachytene, and diplotene stages of prophase I.

**Fig 8:**
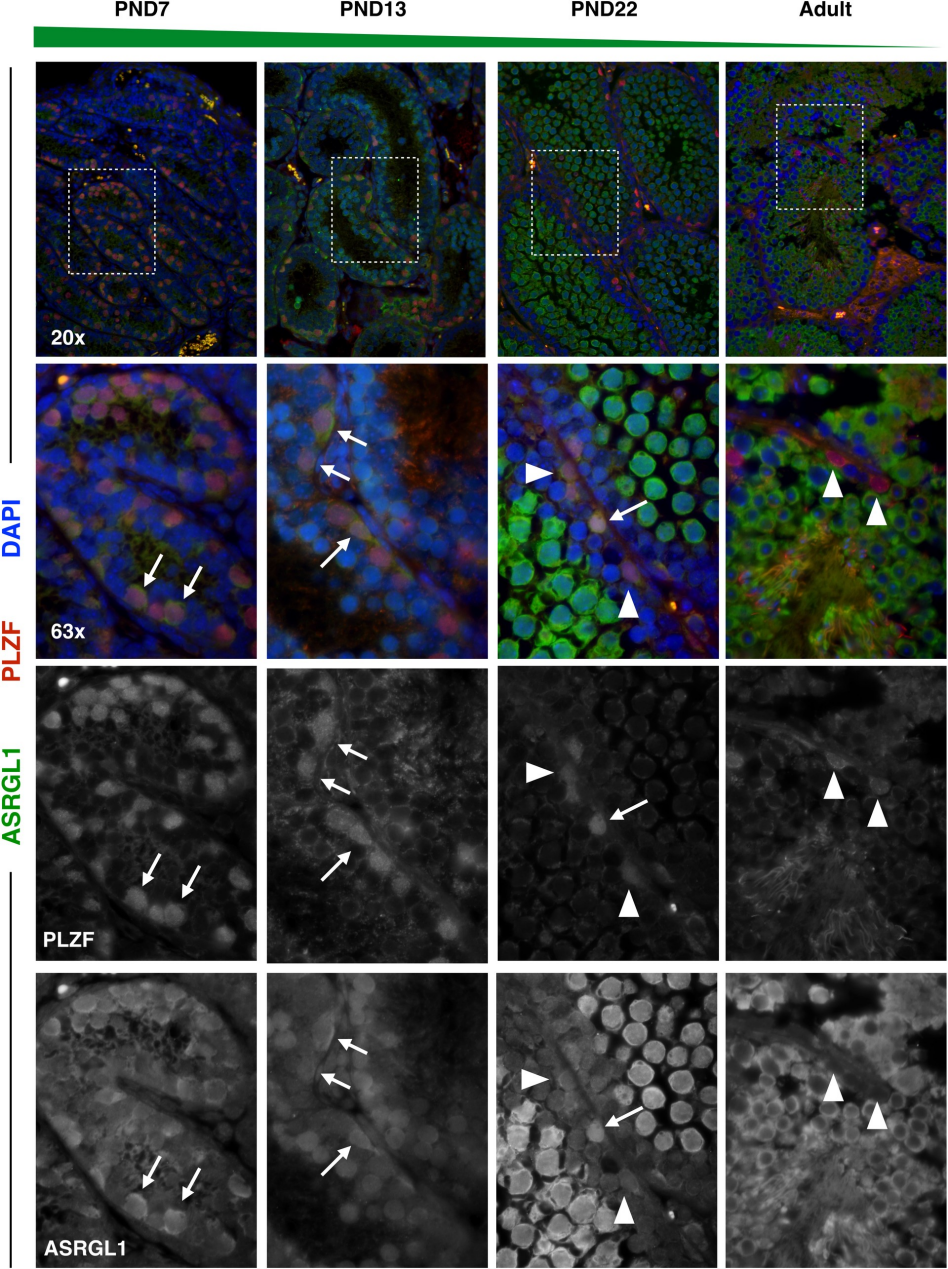
ASRGL1 is highly expressed specifically in spermatogonia from neonatal and juvenile mice. Spermatogonial marker PLZF (red) and ASRGL1 (green) were stained in 5μm testis tissue sections from mice ages PND7, PND13, PND22, and adult. DAPI (blue) denotes nuclei. ASRGL1 protein expression decreases in PLZF+ spermatogonia with age. For all images, high-ASRGL1-expressing spermatogonia are indicated by full arrows with a line, while low-ASRGL1-expressing spermatogonia are indicated by arrowheads.

**Fig 9:**
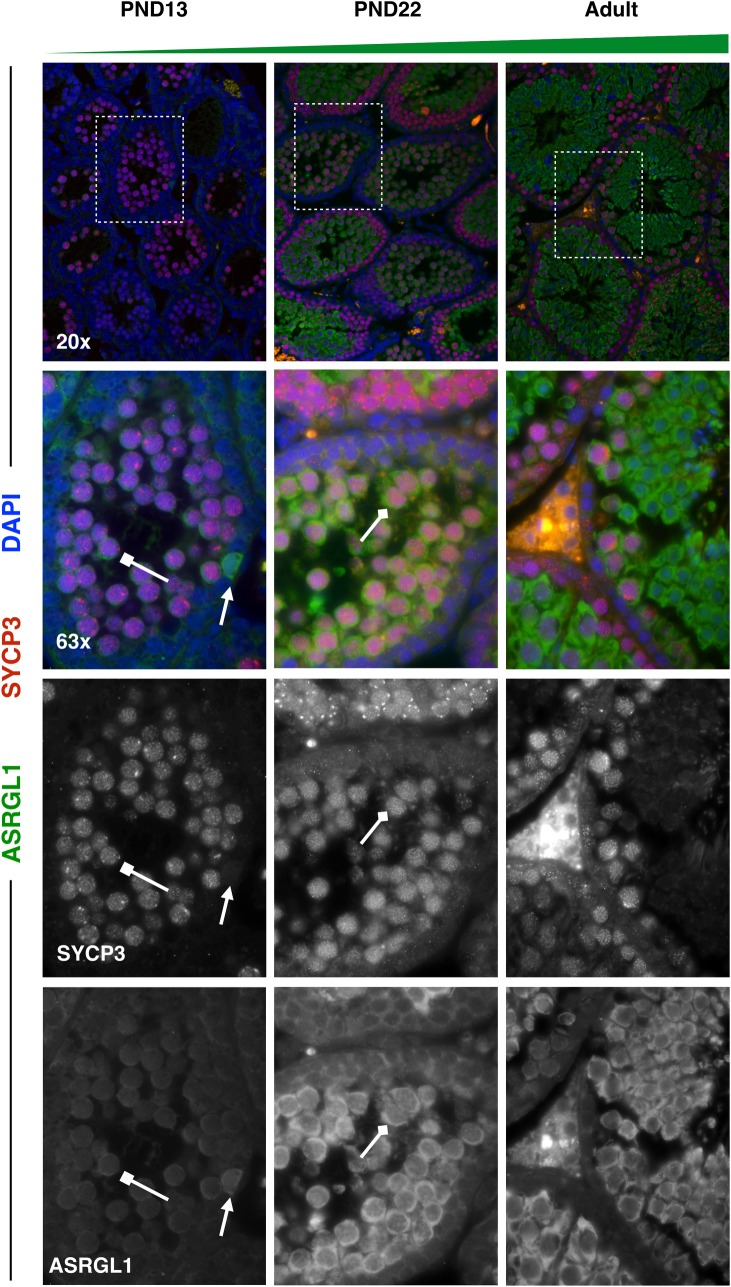
ASRGL1 is highly expressed specifically in spermatocytes of older mice. Spermatocyte marker SYCP3 (red) and ASRGL1 (green) were stained in 5μm testis tissue sections from mice ages PND13, PND22, and adult. ASRGL1 protein expression increases in SYCP3+ spermatocytes with age. For all images, high-ASRGL1-expressing spermatogonia are indicated by full arrows with a line. High-ASRGL1-expressing spermatocytes are indicated by diamond-headed arrows, while low-ASRGL1-expressing spermatocytes are indicated by square-headed arrows.

To profile a range of biological functions including metabolism, enzyme ‘Asparaginase-Like 1’ (ASRGL1; aka ALP1) was chosen for immunofluorescence analysis. ASRGL1 is known to catalyze the hydrolysis of L-asparagine [57] and to clear protein-damaging isoaspartyl-peptides [58], and while largely uncharacterized in the mouse, has been found to be highly expressed in the human cervix, fallopian tube, ovary, and testis [59]. Interestingly, ASRGL1 has been identified as a biomarker of endometrial cancer [59–62], as well as an antigen in rodent sperm [63]. In the single-cell sequencing dataset, *Asrgl1* was observed to be highly expressed in spermatogonia from PND6 mice, with decreasing expression in this cell type in older mice (**Fig 4, S4 Table**). Interestingly, *Asrgl1* was also shown to have dynamic expression in spermatocytes, with the lowest expression detected in PND14 spermatocytes and increasing expression in spermatocytes of older mice (**Fig 6, S6 Table**). These results at the mRNA level were corroborated by immunofluorescence data showing high expression of ASRGL1 protein in PND7 PLZF+ spermatogonia, with decreasing expression of the protein in PND22 and adult PLZF+ spermatogonia (**Fig 8**). Furthermore, PND13 first-wave pachytene spermatocytes expressed little ASRGL1 protein, with expression becoming abundant in pachytene spermatocytes from PND22 and adult testes (**Fig 9**).

Significant differences in RNA stability and processing genes were also observed in spermatogonia during postnatal testis development, with down-regulation of related pathways over time. The RNA binding protein ‘RNA Binding Motif Protein, X-linked-like 2’ (RBMXL2; aka HNRNPG-T) is a putative RNA regulator and splicing factor highly expressed in the mouse testis, specifically in germ cells [64], with critical functions in spermatogenesis [65]. Furthermore, disruptions in RBMXL2 expression and localization in human testes are associated with azoospermia in men [66]. In the single-cell data set, *Rbmx2* mRNA was observed to be highly expressed in spermatogonia from the youngest mice, then decreasing with age (**Fig 4, S4 Table**), with expression persisting in spermatocytes at all ages. Immunofluorescence of RBMXL2 protein demonstrated high expression of the protein in all germ cells, including spermatogonia and spermatocytes. Close inspection, however, revealed relatively higher expression of RBMXL2 in PND7 PLZF+ spermatogonia compared to later time points, despite the relatively consistent expression of RBMXL2 protein in all other germ cell stages at all mouse ages (**S10 Fig**).

**Fig 10:**
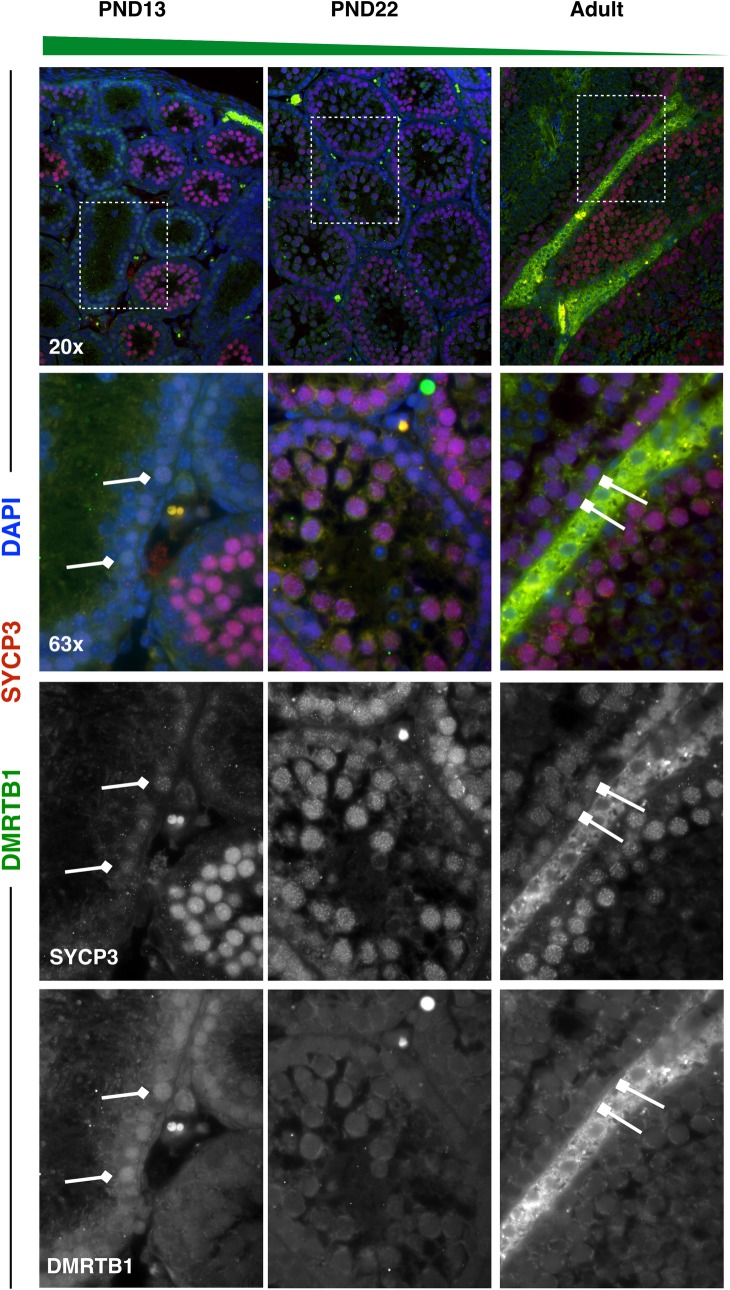
DMRTB1 is highly expressed specifically in first-wave spermatocytes from juvenile mice. Spermatocyte marker SYCP3 (red) and DMRTB1 (green) were stained in 5μm testis tissue sections from mice ages PND13, PND22, and adult. DMRTB1 protein is expressed in the nucleus of first-wave spermatocytes at PND13, with decreasing expression in pachytene spermatocytes with age. For all images, high-DMRTB1-expressing spermatocytes are indicated by diamond-headed arrows, while low-DMRTB1-expressing spermatocytes are indicated by square-headed arrows.

‘Double-sex and Mab-3 Related Transcription Factor B1’ (DMRTB1; aka DMRT6) is a transcriptional regulator known to coordinate the developmental transition from spermatogonial differentiation to meiotic entry [67]. As has been previously observed, *Dmrtb1* mRNA was highly expressed in spermatogonia and early spermatocytes (**Figs 4 & 6**), which we confirmed at the protein level by immunofluorescence. At PND13, first-wave early leptotene spermatocytes, evidenced by spotty SYCP3, exhibited nuclear DMRTB1 staining, while pachytene spermatocytes from all mouse ages lost DMRTB1 expression. Interestingly, the nuclear staining in leptotene spermatocytes was only observed at the earliest time point, PND13, and not seen in early Prophase I spermatocytes of later spermatogenic waves (**Fig 10**).

**Fig 11:**
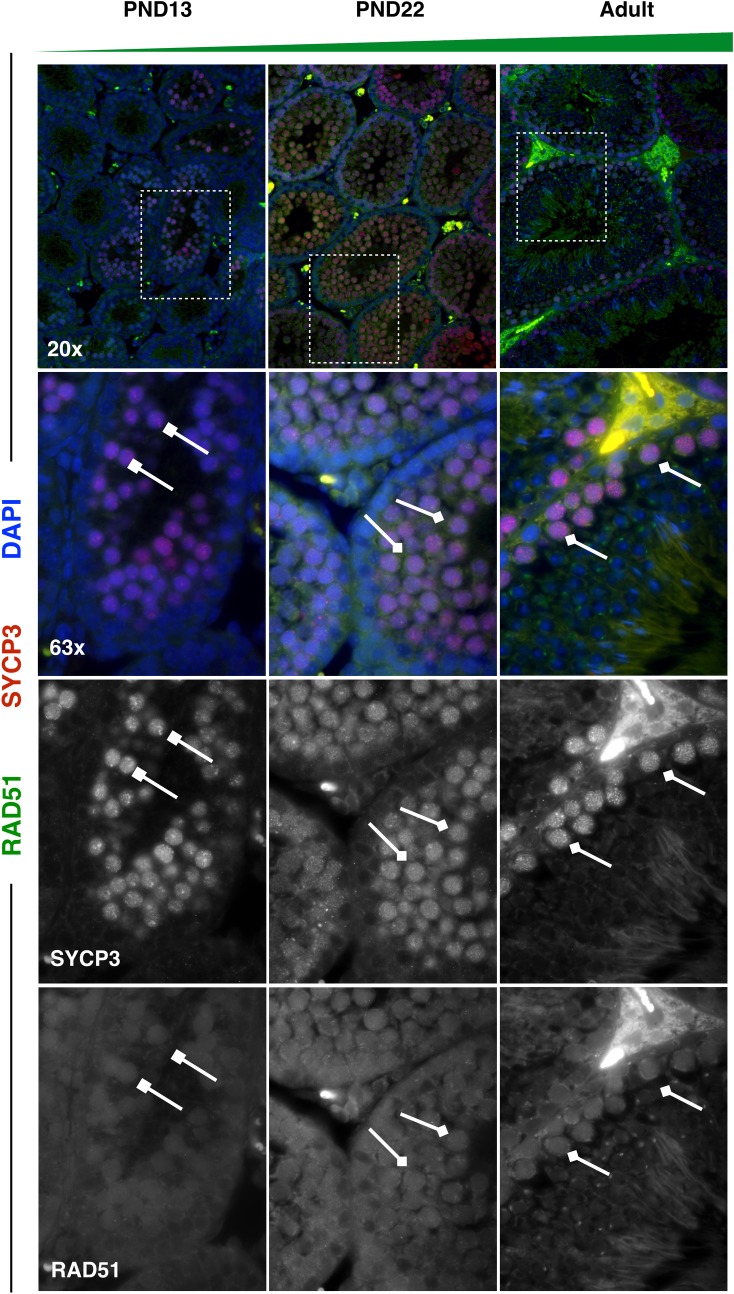
RAD51 has overall reduced protein expression in first-wave spermatocytes from juvenile mice. Spermatocyte marker SYCP3 (red) and RAD51 (green) were stained in 5μm testis tissue sections from mice ages PND13, PND22, and adult. RAD51 protein expression increases in SYCP3+ spermatocytes with age. For all images, high-RAD51-expressing spermatocytes are indicated by diamond-headed arrows, while low-RAD51-expressing spermatocytes are indicated by square-headed arrows.

**Fig 12:**
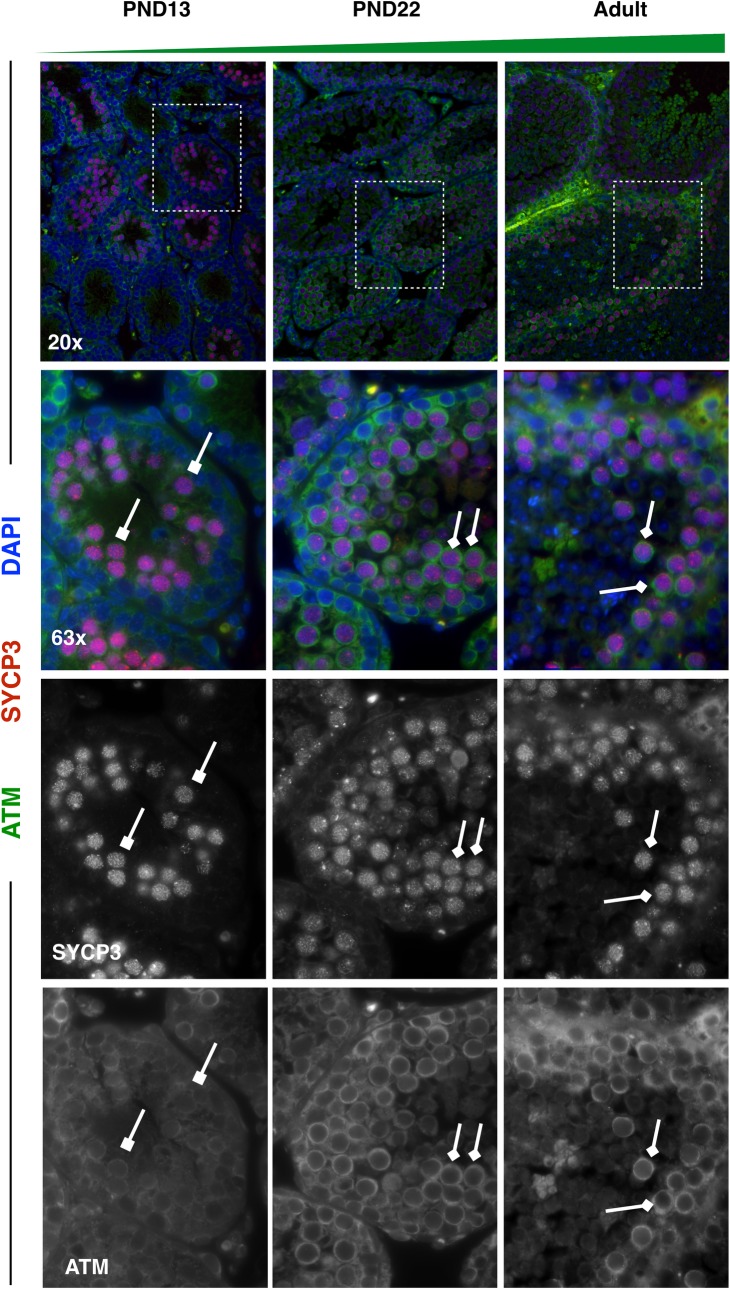
ATM has reduced protein expression in first-wave spermatocytes from juvenile mice. Spermatocyte marker SYCP3 (red) and ATM (green) were stained in 5μm testis tissue sections from mice ages PND13, PND22, and adult. ATM protein expression increases in SYCP3+ spermatocytes with age. For all images, high-ATM-expressing spermatocytes are indicated by diamond-headed arrows, while low-ATM-expressing spermatocytes are indicated by square-headed arrows.

**Fig 13:**
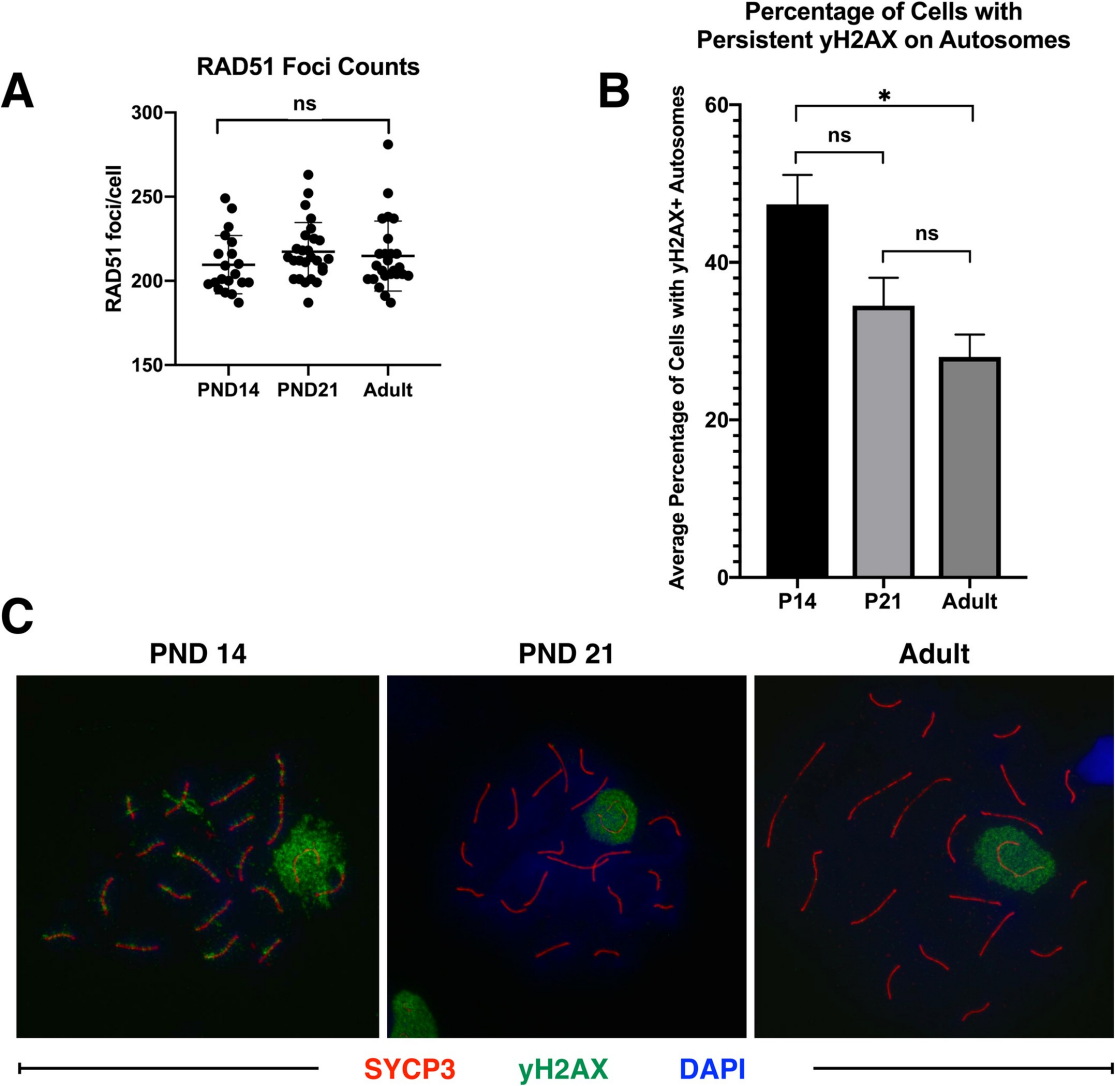
First-wave spermatocytes have persistent autosome-localized γH2AX, but no change in RAD51 foci. A) Meiotic chromosome spreads were prepared from PND14, PND21, and adult mice, and stained for SYCP3 (red) and RAD51 (green). RAD51 foci were quantified per zygotene-staged cell (n = 2 mice per age, with greater than 30 cells quantified). RAD51 foci are not significantly different at any of the profiled ages (Kruskal-Wallis test). B) Meiotic chromosome spreads were prepared from PND14, PND21, and adult mice, and stained for SYCP3 (red) and γH2AX (green). Persistent γH2AX on autosomes was quantified per pachytene-staged cell (n = 2 mice per age, with greater than 30 cells quantified). The average percentage of cells with at least one persistent γH2AX flare on autosomes is significantly higher in first-wave PND14 spermatocytes than in adult spermatocytes (* = p<0.05, One-Way Anova). C) Representative images of pachytene spermatocytes used to produce the quantification in (B).

Finally, DNA damage response proteins ‘RAD51 Recombinase’ (RAD51) and ‘Ataxia Telangiectasia Mutated’ (ATM) were profiled across spermatocytes from mice of increasing age, as these represent particularly interesting candidate proteins whose differential expression may be crucial to understanding aberrant recombination rates and chromosome segregation in the first wave. Intriguingly, both RAD51 and ATM showed subtly, but decidedly, decreased overall protein expression in first-wave pachytene spermatocytes, with increasing protein expression as mice age (**Figs 11 and 12**), as predicted from our scRNAseq data. Previous research has demonstrated significantly fewer RAD51 foci along chromosome cores of juvenile C57bl/6 spermatocytes compared to those at 12 weeks of age [19]. To test if this phenomenon is also observed in B6D2F1/J mice, RAD51 foci were quantified on zygotene chromosome cores in PND14, PND21, and adult spermatocytes. Unlike the differential foci counts observed in C57bl/6 mice, B6D2F1/J mice do not exhibit significant differences in RAD51 foci as a function of age (**Fig 13A**). Our RAD51 observations, therefore, demonstrate overall decreased protein expression in the nucleus of pachytene first-wave spermatocytes, with increasing expression by 3 weeks of age, but no alteration in localized RAD51 during zygonema (**Fig 11**). A similar expression dynamic was observed for ATM, which has robust cytoplasmic staining as well as diffuse nuclear staining in pachytene spermatocytes [68]. Our data demonstrate decreased expression of ATM in both cellular compartments in the first-wave spermatocytes, with increasing expression, particularly in the cytoplasm of these cells, by 3 weeks of age (**Fig 12**).

To further dissect DNA damage response in first-wave spermatocytes, γH2AX staining was performed on chromosome spreads from PND14, PND21 and adult spermatocytes (**Fig 13B and 13C**). Canonically, the γH2AX histone variant marks meiotic double-strand breaks (DSBs) [69] in leptonema and zygonema, becoming restricted to the unsynapsed “sex body”, or XY chromosomes, during pachynema [33]. Presence of γH2AX on pachytene autosomes indicates aberrant unrepaired DSBs. Quantification of γH2AX demonstrated a significantly greater average percentage of cells with persistent flares on pachytene autosomes compared to adult spermatocytes (**Fig 13B**). These age-dependent dynamics of critical DNA damage response regulators are likely to contribute to the health and viability of resulting spermatocytes and spermatozoa from these spermatogenic cycles, and may underlie some of the functional differences observed in the first wave of spermatogenesis [6,19,20].

Overall, these gene expression dynamics discovered from single-cell mRNA sequencing are reproducible at the level of protein expression in the context of the native tissue, and likely represent important transitions both in spermatogonia and spermatocytes during testis maturation. These data will be indispensable to investigate how gene expression dynamics help to regulate the many critical developmental events, including spermatogonial differentiation and meiotic progression, occurring in the developing mouse testis.

## Discussion

We have performed the first comprehensive sampling and screening of mouse germ cells from neonatal life through adulthood, to characterize transcriptional profiles at single-cell resolution. With the exception of the adult samples, which were sorted to minimize the over-riding sperm component, all libraries were generated from unsorted single-cell suspensions containing all testicular cells. Adult samples were minimally processed with a single-step magnetic cell sort to provide representation of all germ cell types in the adult testis. Previously, single-cell-sequencing studies on sorted cells have provided valuable information about specific, and marker-defined cell types [15,70]. The study presented here, however, focuses on profiling the germline during postnatal testis maturation, importantly capturing the first-wave of spermatogonia and spermatocytes which exhibit differences from later, steady-state spermatogenesis. Because of the progression of ages profiled, we have captured changes in gene expression at single-cell resolution to compare the developmental progression of spermatogenesis as mice age.

Germ cells subtypes in the testis are frequently defined by the presence or absence of particular protein markers, which can be visualized by reporter expression or immunofluorescence, or may be used for flow cytometry or other enrichment paradigms. Spermatogonia are often defined as cells which express a key complement of protein markers, such as PLZF or ‘GDNF family receptor alpha 1’ (GFRα1) [71–73]. While this is the best practice for visual identification of cells for which discrete markers have been elusive, such as spermatogonia, our analysis suggests that the biology of these cells during postnatal testis development is far more complicated than previously understood. Our analysis stresses that defining these cell populations on the basis of specific markers may be overly simplistic, despite being the current standard practice in the field. Primary spermatocytes similarly show age-specific patterns at the transcriptional level. Furthermore, cells possessing an SYCP3-positive synaptonemal complex indicative of pachynema also exhibit differences in immunolocalized proteins during testis maturation, indicating that they, too, exhibit distinct and variable expression patterns with increasing age. Critically, this analysis reveals that, while known markers may be useful for defining primary cell identity, there are many changes in spermatogenesis during mouse development that have been under-appreciated without the power of single-cell resolution of gene expression profiling.

Specifically, we show here that PLZF-defined spermatogonia, though retaining PLZF-positivity, are transcriptionally distinct at PND6 compared to later developmental time points. These transcriptional dynamics are also reflected by distinct differences at the protein level, with proteins such ASRGL1 being localized strongly in PLZF+ spermatogonia during the first weeks of life, but decreasing in expression in these cells around three weeks of age (**Fig 8**). Similarly, we show that Prophase I spermatocytes possess significant transcriptional differences in the first-wave compared to subsequent spermatogenic waves, with hundreds of differentially expressed genes across multiple regulatory pathways. Furthermore, direct inspection of pachytene spermatocytes from the first wave at PND13 to later spermatogenic waves reveals that, like spermatogonia, transcript dynamics are also reflected at the protein level. For example, proteins such as DMRTB1 are found only in the nuclei of first-wave leptotene spermatocytes as they transition from differentiated spermatogonia into the meiotic program, but not in spermatocytes from older mice (**Fig 10**). By contrast, other proteins such as ASRGL1 are in low abundance in early first-wave spermatocytes, but become more strongly immunolocalized in spermatocytes at increasing ages (**Fig 9**).

In addition to the transcriptional and translational dynamics in defined cell types, these data also reveal differential utilization of particular biological pathways over developmental time. Gene set enrichment analysis [37] utilizing the Reactome pathway database [38,39] has demonstrated that spermatogonia significantly change their transcriptional landscape as mice age, including downregulation of genes within essential meiotic-entry-associated SCF/KIT [43,74] and FGFR [75] pathways, including *Kit* and *KitL* (**S9A Fig**). While we cannot rule out the possibility that variable gene expression in spermatogonia is, in part, due to differential contributions of the spermatogonial stem cell population at different ages–with decreasing contribution as mice age–this dataset provides strong support for true variable gene expression in the spermatogonial pool. For instance, while spermatogonia derived from older mice exhibit downregulation of genes associated with FGFR signaling, including *Fgf8* and *Fgfr1*, and could indicate decreased representation of an undifferentiated spermatogonial population [45], this is in opposition to observed coincident decreased expression of *Kit* and *KitL* which would support increased representation of an undifferentiated population [41,43,74] (**S9A Fig**). It is interesting to consider that these data may reflect overall changes to the paracrine signaling of the spermatogonial stem cell niche as well as the basement membrane as mice age. Overall, these data suggest spermatogonia may modulate their sensitivity for particular critical signaling pathways, which may affect their competency to commit to the meiotic program. Furthermore, pathways associated with mRNA stability and protein degradation are upregulated as the testis matures, suggesting that spermatogonia from older mice may change their capacity for post-transcriptional and post-translational regulation, possibly reflecting changing demands for growth and proliferation in older animals.

Similar to spermatogonia, spermatocytes also exhibit differential utilization of specific biological pathways with age, an observation that dovetails with the knowledge that spermatocytes derived from the first wave of spermatogenesis are functionally different to those spermatocytes derived from steady-state spermatogenesis. First-wave spermatocytes are known to exhibit several unique, and some detrimental, characteristics, including reduced recombination rate [19,20] and greater incidence of chromosome mis-segregation [20]. These features may help to explain some of reproductive deficits observed in spermatozoa derived from young fathers compared to older fathers [76,77]. Our data presented here demonstrate age-related upregulation of pathways associated with DNA replication and repair, double strand break repair, and response to DNA damage (**S9B Fig**), all of which may underlie the well-characterized differences between spermatocytes in the first wave compared to steady-state spermatocytes. Included in these sets of variably-expressed genes are known regulators of DNA damage response and cross-over formation including *Rad51* [50], *Brip1* [46], *and H2afx* [51], as well as *Brca1* and *Brca2* [47–49] and *Atm* [52,78], all of which increase in spermatocytes from older mice, effects which we have also shown at the protein level for both RAD51 and ATM (**Figs 11 and 12**). While the cause of this lower expression in the first wave is unknown, we have also shown that it is coincident with persistent γH2AX on autosomes during pachynema (**Fig 13**), pointing to systemic alterations in the processing of DNA damage during the initial waves of spermatogenesis. This interpretation is further supported by work from Lange et al, in which the authors find that reduced ATM expression results in increased DSBs [78]. These observations may suggest that steady-state spermatocytes acquire greater competency to cope with the DNA damage inherent to meiotic progression, and that spermatocytes in the first wave may not execute these pathways as successfully, resulting in the observed recombination differences and increased chromosome mis-segregation. Moreover, these early meiotic waves in boys are thought to be highly error-prone and thus these data may provide an explanation for the increased aneuploidy observed in the progeny of young fathers [76,77]. Notably, like spermatogonia, spermatocytes also experience alteration of pathways related to translation and mRNA stability, emphasizing the myriad ways in which gene expression is regulated in developing germ cells. Ultimately, this differential pathway utilization may help to explain not only the functional differences observed in spermatocytes and spermatozoa from juveniles, but may also improve our understanding of increased birth defects associated with young paternal age [76,77].

An important consideration in these studies is the changing architecture during the maturation of the murine testis, which spans the time points we have chosen for study. Between two to three weeks of age in the rodents [79], the seminiferous epithelium becomes segregated into basal and adluminal compartments due to the formation of the blood-testis barrier (BTB), involving the establishment of a variety of cell-cell junctions between Sertoli cells (reviewed comprehensively in Mruk & Chang [80]). The BTB allows for the physical separation between spermatogonia and pre-leptotene spermatocytes in the basal compartment from all more differentiated spermatocytes and spermatogenic cell types in the adluminal compartment. Functionally, the BTB allows for meiotic cells to be maintained and matured in an immune-privileged environment, disconnected from the circulatory system of the animal [81]. As a result of this maturation of the seminiferous epithelium and the cyclicity of spermatogenesis, germ cell groups can synchronously progress through spermatogenesis longitudinally through the tubule, resulting in the many germ cell stages of the seminiferous epithelial cycle [82]. Due to the formation of these structures taking place in the time frame between our PND6/14/18 libraries and our PND25/30/adult libraries, we cannot rule out the possibility that some of the differences we observe in first-wave spermatocytes are due to their maturation in a seminiferous tubule environment that has not yet established this architecture, or due to their different stages in the epithelial cycle. There is likely complex interplay between the maturation of the environment within the tubule, the cell-cell signaling taking place, and the resulting changes in biological pathway utilization in the first-wave spermatocytes compared to those in steady-state spermatogenesis.

An additional primary objective in undertaking this analysis was to potentially reveal new markers of the spermatogonial stem cell population. Despite many efforts to define this population by both cytoplasmic and nuclear markers, discrete markers of this population have remained elusive and controversial [83–86]. Our data are supportive of the high degree of heterogeneity of this cell population, not only within the population at a single age, but also across ages. Furthermore, candidate cell-type specific markers which have become accepted in the field, such as expression of ‘Inhibitor of DNA Binding 4’ (*Id4*) [87–89], show widespread detection in spermatogonia through spermatocytes, while markers such as *Zbtb16* (*Plzf*) and *Gfr*α*1* have much more restricted expression (**S6 Fig**). Importantly, expression patterns of these popular markers are not entirely self-consistent. It is therefore likely that the spermatogonial population is not discrete, but is indeed a continuous or plastic population [90,91]. These data represent a valuable resource to the study of the molecular mechanisms underlying SSC self-renewal and differentiation, though the biology may not be as simplistic as originally thought.

Taken together, these data represent the first comprehensive sampling and profiling of spermatogonia and spermatocytes during development of the mouse testis. These data emphasize the necessity of considering not only the protein markers for which individual cells are positive, but also the age of the cells being analyzed. These observations of dynamic gene expression in germ cell populations during postnatal testis development stress that germ cells of a particular age or identity possess distinct profiles and that consideration must be given to these dynamics when profiling germ cell populations. These data also represent an invaluable community resource for discovery of previously unknown gene expression dynamics and pathway contributions that may be critical for the many developmental transitions in the male germ cell population which are essential for successful spermatogenesis and fertility.

## Methods

### Ethics statement

Work involving laboratory rodents was performed under an approved protocol, 2004–0063, from the Cornell Institutional Animal Care and Use Committee. Mice were maintained on standard conditions of light:dark cycling and temperature, with *ad libitum* access to rodent chow and water.

### Animals

B6D2F1/J mice were generated by mating C57bl/6 female mice with DBA/2J male mice. All animal protocols were reviewed and approved by the Cornell University Institutional Animal Care and Use Committee and were performed in accordance with the National Institutes of Health Guide for the Care and Use of Laboratory Animals. Mice were maintained on standard light:dark cycles with laboratory mouse chow provided *ad libidum*.

### Generation of testis single cell libraries

Testes were collected from mice (n = 1 mouse, 2 testes for each time point) at postnatal (PND) days 6, 14, 18, 25, 30, and 8 weeks of age, and dissociated per standard protocols for germ cell enrichment [21]. Briefly, testes were held in 1X HBSS buffer before de-tunicating and moving tubules into 0.25% Trypsin. Tubules were further dissociated by trituration and addition of DNase to a final concentration of 7 mg/ml. Tubules were placed in a 37°C incubation for 5 minutes at a time, and then removed for further trituration. Incubations at 37°C were performed three times, until a cloudy suspension was achieved. Cells were passed through a 40 μM filter, spun down, and re-suspended in 10ml 1X Dulbecco’s PBS + 10% Knockout Serum Replacement (DPBS-S). This cell suspension was then layered on top of a 30% Percoll solution. Cells were then spun down again, and the resulting pellet was re-suspended in 1ml DPBS-S. As a technical control, cells from PND18 were split into two samples after the 40 μM filter, with one half of the cells processed with the Percoll gradient, and the other half directly re-suspended in its final buffer with no Percoll sedimentation, resulting in libraries “PND18” and “PND18pre”, respectively. Due to the similarities between these libraries (**S1 Fig**), the data from these libraries were thereafter combined and analyzed together as “PND18”.

For adult testes only, the resulting cell suspension was split in half and sorted with magnetic beads in two ways: (1) sperm-depletion was performed by incubating the cells for 30 minutes with 20 μl anti-ACRV1-PE (Novus Biologicals #NB500-455PE), washing with DPBS-S, incubating the cells for 30 minutes with 20 μl magnetic-bead-conjugated anti-PE (Miltenyi Biotec #130-048-801), and finally performing a negative magnetic selection. Cells were applied to a Miltenyi Biotec MACS LS column, and flow-through cells were collected, as sperm were to remain bound to the ferromagnetic column. (2) THY1+ spermatogonia were enriched by incubating the cells for 60 minutes with 20 μl magnetic-bead-conjugated anti-CD90.2 (THY1) (Miltenyi Biotec #130-102-464), and finally performing a positive magnetic selection. Cells were applied to the column, flow-through cells were discarded, and antibody-bound cells were eluted from the ferromagnetic column. These cells were then spun down and re-suspended in 1ml DPBS-S as above.

The resulting cells from all samples were submitted to the Cornell DNA Sequencing Core Facility for processing on the 10X Genomics Chromium System with a target of 4–5000 cells per sample. Sequencing libraries were generated using the 10X Genomics Chromium Single Cell 3′ RNAseq v2 kit, tested for quality control on an ABI DNA Fragment Analyzer, and run on a NextSeq platform with 150 base-pair reads. Libraries were sequenced to an average depth of 98M reads (range 77M-124M); on average, 91% of reads (range 89%-92%) mapped to the reference genome.

### Single-cell transcriptome analysis

Data normalization, unsupervised cell clustering, and differential expression were carried out using the Seurat R package [92]. Batch effect and cell-cycle effect were removed by Combat [93] and Seurat together. Cells with less than 500 genes or 2000 UMIs or more than 15% of mitochondria genes were excluded from the analysis. Gene expression raw counts were normalized following a global-scaling normalization method with a scale factor of 10,000 and a log2 transformation, using the Seurat NormalizeData function. The top 4,000 highly variable genes were selected using the expression and dispersion (variance/mean) of genes. Combat removed batch effects. Seurat regressed the difference between the G2M and S phase, then followed by principal component analysis (PCA). The most significant principal components (1–30) were used for unsupervised clustering and t-Distributed Stochastic Neighbor Embedding (tSNE) analysis.

Cell types were manually identified by characteristic marker genes [18,94,95], and confirmed by SingleR (Single-cell Recognition) package. Differential expression analysis was performed based on the MAST (Model-based Analysis of Single Cell Transcriptomics) [36]. Gene Set Enrichment Time Series Analysis [37] used the differential expression based on each time point, after removing genes highly expressed in spermatids (**S7 and S8 Figs**). Pathways were visualized by EnrichmentMap [39] in Cytoscape [38].

Code availability: The scripts used for analysis and figure generation are available at https://github.com/nyuhuyang/scRNAseq-SSCs

Data availability: The single-cell RNAseq data have been deposited at GEO and are accessible through Series accession number GSE121904.

### Immunofluorescence validation

Testes were collected at PND7, PND13, PND22, and 8 weeks of age, cleaned of excess fat, and fixed in 0.1% formalin solution overnight before dehydration and embedding in paraffin. Note that for these experiments, while we used the same time frames for scSEQ samples representing the range of testis maturation from SSC specification through the first wave and into steady-state spermatogenesis, the precise days of collection were dependent on the simultaneous harvesting and processing of samples from all age groups to reduce batch effects and allow well-controlled comparisons among ages.

Fixed testes were sectioned at 5 μm onto glass slides by the Cornell Animal Health Diagnostic Center. To stain, sections were de-paraffinized by 3x, 5 minute washes in Histoclear followed by rehydration in 100% ethanol (2x, 5 minutes), 95% ethanol (2x, 5 minutes), 70% ethanol (1x, 5 minutes), water (1x, 5 minutes). Sections were then incubated in boiling antigen retrieval buffer (10 mM sodium citrate, 0.05% Tween-20, pH 6.0) for 20 minutes and left to cool. Sections were washed 3x, 5 minutes in 1X PBS + 0.1% Triton-X (PBST). Tissue sections were then incubated in blocking buffer [3% Goat Serum (Sigma), 1% Bovine Serum Albumin (Sigma), and 0.5% Triton-X (Fisher Scientific) in 1X PBS] and stained by incubation with primary antibodies against PLZF, SYCP3, RBMXL2, ASRGL1, DMRTB1, RAD51, ATM, and γH2AX (see **S8 Table**) overnight at 4°C. The following day, slides were washed 3x, 5 minutes in PBST and then incubated with secondary antibodies raised in goat against mouse (594 nm) and rabbit (488 nm) at 1:500 for 1 hour at 37°C. A secondary antibody-only control was included to assess background staining. Sections were further stained with DAPI to visualize nuclei, mounted and analyzed on an Epifluorescent Zeiss Axioplan microscope. For a given set of antibodies, images were exposed equivalently for all samples from different time points to generate images for relative comparison of intensity over time.

### Meiotic chromosome analysis

Spreading of meiotic chromosomes was performed using the “drying down method” as previously described [96]. Briefly, testes were simultaneously collected from mice aged 14 days, 21 days, or 8 weeks, de-tunicated, and incubated in hypotonic extraction buffer for one hour on ice. Tubules were then minced in a bubble of 0.03% sucrose and the cell suspension applied to 1% paraformaldehyde wetted slides. Cells were allowed to “spread” for two hours in humidity, followed by drying. Slides were subsequently stained with primary antibodies against either RAD51 or γH2AX in conjunction with primary antibody against SYCP3 (see **S8 Table**). Secondary antibodies used are the same as those used for testis section immunofluorescence. For analysis of RAD51 foci, SYCP3 was used to identify zygotene spermatocytes. Two animals per age and at least 30 cells per mouse were analyzed for number of RAD51 foci on the chromosomes cores per cell. Significance was determined by Kruskal-Wallis test. For analysis of persistent γH2AX, SYCP3 was used to identify pachytene spermatocytes. Two animals per age and at least 30 cells per mouse were analyzed for presence or absence of γH2AX on the autosomes. Significance was determined by a one-way Anova.

## Supporting information

S1 Fig Similarities between “PND18pre” and “PND18” libraries. A) Germ and somatic cell composition by proportion and absolute cell number from libraries “PND18pre” (pre-Percoll) and “PND18” (post-Percoll). B) tSNE representation of all cells with >500 detected genes and >2000 UMIs. PND18 libraries are color-coded while other libraries are greyed out. C) Cell counts for each cell type plotted in (A). As a result of all of these similarities, the data derived from the libraries was combined for analysis.
(TIF)

S2 Fig Quality control violin plots of single-cell data before and after filtering. A) Distribution of gene and UMI counts, and mitochondrial gene percentage per library-of-origin, before and after data filtration. “Ad” indicates adult libraries, with ACRV1-D indicating ACRV1+ depletion, while THY1-E indicates THY1+ enrichment. B) Distribution of gene and UMI counts, and mitochondrial gene percentage per cell type. UMI = “unique molecular identifier”, used to count unique transcripts.
(TIF)

S3 Fig Computationally determined unsupervised cell clusters. Representative clustering of all cells with >500 detected genes and >2000 UMIs, based on most significant principal components, color-coded by cell cluster.
(TIF)

S4 Fig Dot plot analysis of Sertoli cell marker genes and X-linked genes. A) Dot plot representation of immature and mature Sertoli cell marker genes per cell cluster as determined in **S3 Fig**. Canonical immature Sertoli cell markers include *Amh* and *Dhh*, while mature markers include *Gata1* and *Clu* (*Spg-2*). *Wt1* and *Sox9* are markers of all Sertoli cells [25–28]. Notably, for mature Sertoli cell marker genes which are only detected in a small percentage of cells in the cluster (as indicated by dot size), including *Fshr* and *Gata1*, expression in those cells is quite high as indicated by the dot’s red color. B) Dot plot representation of a sampling of X-linked genes per cell cluster as determined in **S3 Fig**. Four general patterns of expression are apparent: genes denoted by the asterisk (*) are detected robustly in spermatogonia, spermatids, and several types of somatic cells. Genes denoted by an ampersand (&) are detected robustly in spermatogonia and round spermatids. Genes denoted by the pound sign (#) are detected robustly in spermatogonia and several types of somatic cells. Genes denoted by the delta sign (Δ) are detected robustly in spermatogonia only. Genes denoted by a cross (⍏) are not robustly detected in any cell types. Overall, despite the different patterns of expression, spermatocytes have uniformly low detection of X-linked genes, as is expected due to meiotic sex chromosome inactivation.
(TIF)

S5 Fig Dot plot analysis of characteristic marker genes based on cluster and cell type. A) Dot plot representation of known marker genes per cell cluster determined in **S3 Fig**. B) Dot plot representation of known marker genes per cell type (supercluster) determined in **Fig 2B**.
(TIF)

S6 Fig Representative germ cell marker expression in cells from all libraries. tSNE plot of germ cells from all libraries colored by cell type (top), as well as tSNE plots annotated with notable germ cell marker gene expression (bottom).
(TIF)

S7 Fig Detection of spermatid genes in all cell types only in libraries containing spermatids. Representative tSNE plots of A) *Oaz3*, B) *Prm1*, and C) *Prm2*, showing detection of the indicated gene across all cell types in older samples only. Overlaid dotted lines indicate cell cluster identity by color. While minimal detection of example genes is observed in a small percentage of PND18 cells, robust expression does not become apparent until PND25, the first sample in which spermatids are present. Crucially, endogenous expression of these genes is not expected in somatic cell types, and detection of these transcripts in somatic cell types is therefore additional evidence of contaminating cell-free RNA.
(TIF)

S8 Fig Spermatid gene expression in other cell types is likely due to cell-free RNA contamination and not true expression of spermiogenesis genes. Heatmap of filtered spermatid genes per cell type (indicated by color bar), excluding elongated spermatids. Expression is scaled, ranging from 0 to 2, and excludes elongated spermatids. Contaminating spermatid gene detection is observed in somatic and germ cell types only in libraries in which spermatids are present (PND25 and later), suggesting that the detection of these genes is due to lysis of spermatids in the original material, and not premature expression of spermatid genes, which would have likely been observed in PND14 and PND18 as well. As is also observed in **S7 Fig**, endogenous expression of these genes is not expected in somatic cell types—denoted in this heatmap by a red arrow in the adult library—and is therefore additional evidence to support that detection of these transcripts in somatic cell types is due to contaminating cell-free RNA.
(TIF)

S9 Fig GSEA enrichment plots for pathways correlated with spermatogonia and spermatocyte development. A) Enrichment plots for selected Reactome database pathways in spermatogonia. Pathways “SIGNALING_BY_SCF_KIT” and “SIGNALING_BY_FGFR” show negative correlation with developmental time, while pathways “DESTABILIZATION_OF_MRNA” and “SIGNALING_BY_WNT” show positive correlation with developmental time. B) Enrichment plots for selected Reactome database pathways in spermatogonia. All pathways shown demonstrate positive correlation with developmental time.
(TIF)

S10 Fig RBMXL2 is highly expressed specifically in spermatogonia from neonatal and juvenile mice, decreasing with age. Spermatogonial marker PLZF (red) and RBMXl2 (green) were stained in 5μm testis tissue sections from mice ages PND7, PND13, PND22, and adult. DAPI (blue) denotes nuclei. RBMXL2 protein expression decreases in PLZF+ spermatogonia with age. High-RBMXL2-expressing spermatogonia are indicated by full arrows with a line, while low-RBMXL2-expressing cells are indicated by arrowheads. Individual channels are represented in gray scale.
(TIF)

S1 Table Cell counts per library of origin. Cell types were manually identified by marker genes, and confirmed by the SingleR (Single-cell Recognition) package. Cell counts for all germ cell and somatic cell types per library of origin are listed as well as total cells per library of origin.
(XLSX)

S2 Table Genes expressed in specific germ cell types. Marker genes were determined that distinguish different primary cell clusters. Up to 250 genes per primary cell type are listed, with statistics from Seurat comparing expression in the marker-associated cell type (X.1) to all other germ cells (X.2). Data for these genes are depicted as row-normalized gene expression in individual cells in **Fig 3**.
(XLSX)

S3 Table Genes with high counts in spermatids (filtered out). Due to contaminating cell-free mRNA derived from lysed spermatids (detected only in samples in which spermatids are present), these genes expressed at high levels in spermatids were removed from the dataset. The UMI1/UMI2 ratio reflects the expression of each gene in spermatids relative to all other germ cells.
(XLSX)

S4 Table Genes with variable expression in spermatogonia during testis maturation. Model-based analysis of single-cell transcriptomics (MAST) was utilized to identify genes that are variably expressed in spermatogonia as a function of mouse age. Genes listed in this table include “markers” (genes with significantly upregulated expression in all spermatogonia), as well as genes with significant variation in expression in spermatogonia during testis maturation. Data for these genes are depicted as row-normalized gene expression in individual cells in **Fig 4**.
(XLSX)

S5 Table Gene Set Enrichment Analysis of Reactome pathways in spermatogonia during testis maturation. Reactome pathways with significantly differential expression as a function of mouse age in spermatogonia are included (FDR < 0.05); the same results are displayed in Cytoscape diagrams in **Fig 5**. The table includes GSEA results for each significant Reactome pathway, including the gene list, enrichment score (ES), normalized enrichment score (NES), and results of the statistical test for enrichment.
(XLSX)

S6 Table Genes with variable expression in spermatocytes during testis maturation. Model-based analysis of single-cell transcriptomics (MAST) was utilized to identify genes that are variably expressed in spermatocytes as a function of age. Genes listed in this table include “markers” (genes with significantly upregulated expression in all spermatocytes), as well as genes with significant variation in expression in spermatocytes during testis maturation. Data for these genes are depicted as row-normalized gene expression in individual cells in **Fig 6**.
(XLSX)

S7 Table Gene Set Enrichment Analysis of Reactome pathways in spermatocytes during testis maturation. Reactome pathways with significant differential expression as a function of mouse age in spermatocytes are included (FDR < 0.05); the same results are displayed in Cytoscape diagrams in **Fig 7**. The table includes GSEA results for each significant Reactome pathway, including the gene list, enrichment score (ES), normalized enrichment score (NES), and results of the statistical test for enrichment.
(XLSX)

S8 Table Antibodies used for immunofluorescent staining. All primary and secondary antibodies used for immunofluorescence validation are documented with product numbers and dilutions used.
(XLSX)
