# Supplementary figures and images for: Dynamic transcriptome profiles within spermatogonial and spermatocyte populations during postnatal testis maturation revealed by single-cell sequencing
Source: PLoS Genet. 2019 Mar 20;15(3):e1007810. doi: 10.1371/journal.pgen.1007810 (PMC6443194; doi:10.1371/journal.pgen.1007810)

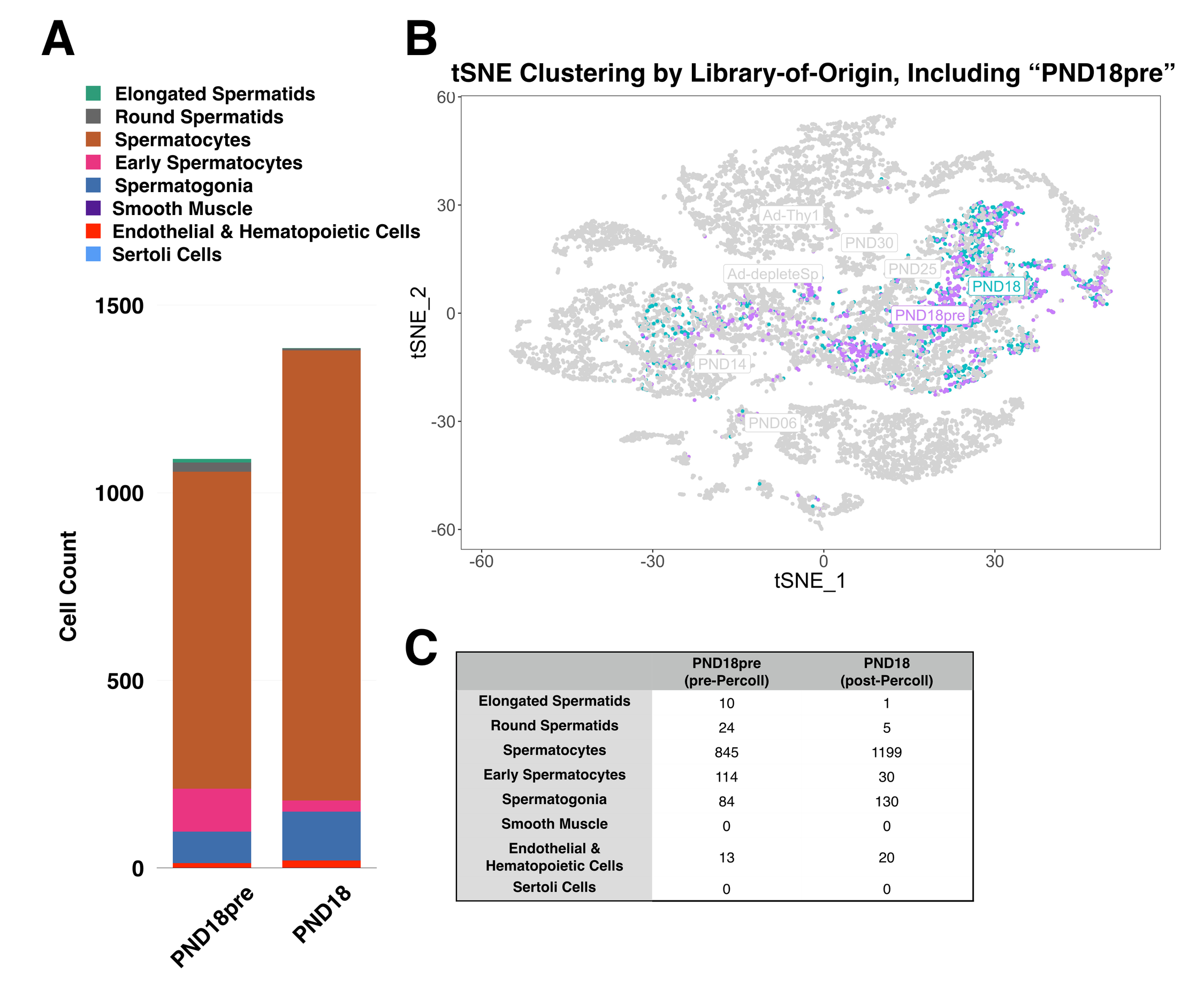

Supplement: S1 Fig — A) Germ and somatic cell composition by proportion and absolute cell number from libraries “PND18pre” (pre-Percoll) and “PND18” (post-Percoll). B) tSNE representation of all cells with >500 detected genes and >2000 UMIs. PND18 libraries are color-coded while other libraries are greyed out. C) Cell counts for each cell type plotted in (A). As a result of all of these similarities, the data derived from the libraries was combined for analysis. (TIF) [file pgen.1007810.s001.tif]

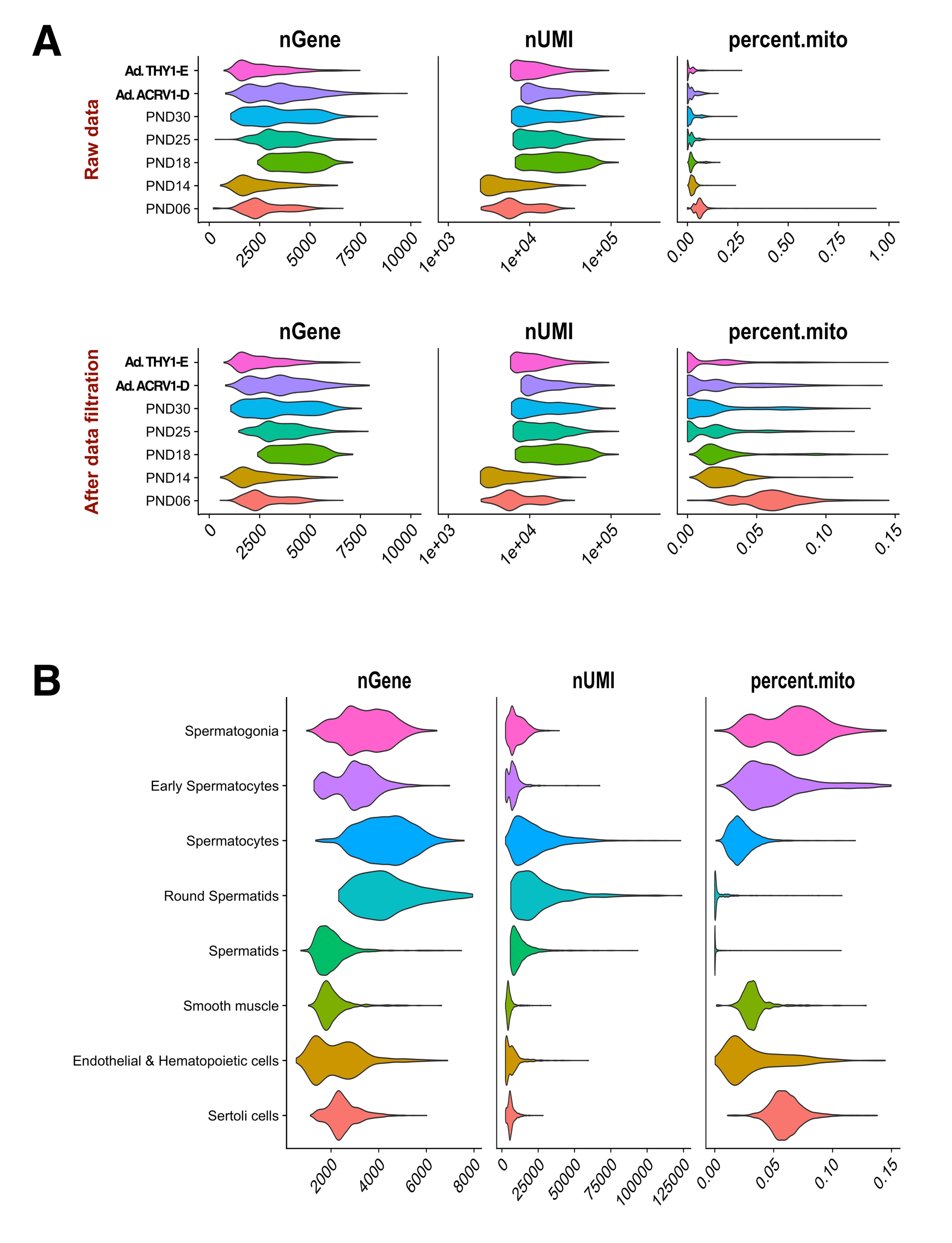

Supplement: S2 Fig — A) Distribution of gene and UMI counts, and mitochondrial gene percentage per library-of-origin, before and after data filtration. “Ad” indicates adult libraries, with ACRV1-D indicating ACRV1+ depletion, while THY1-E indicates THY1+ enrichment. B) Distribution of gene and UMI counts, and mitochondrial gene percentage per cell type. UMI = “unique molecular identifier”, used to count unique transcripts. (TIF) [file pgen.1007810.s002.tif]

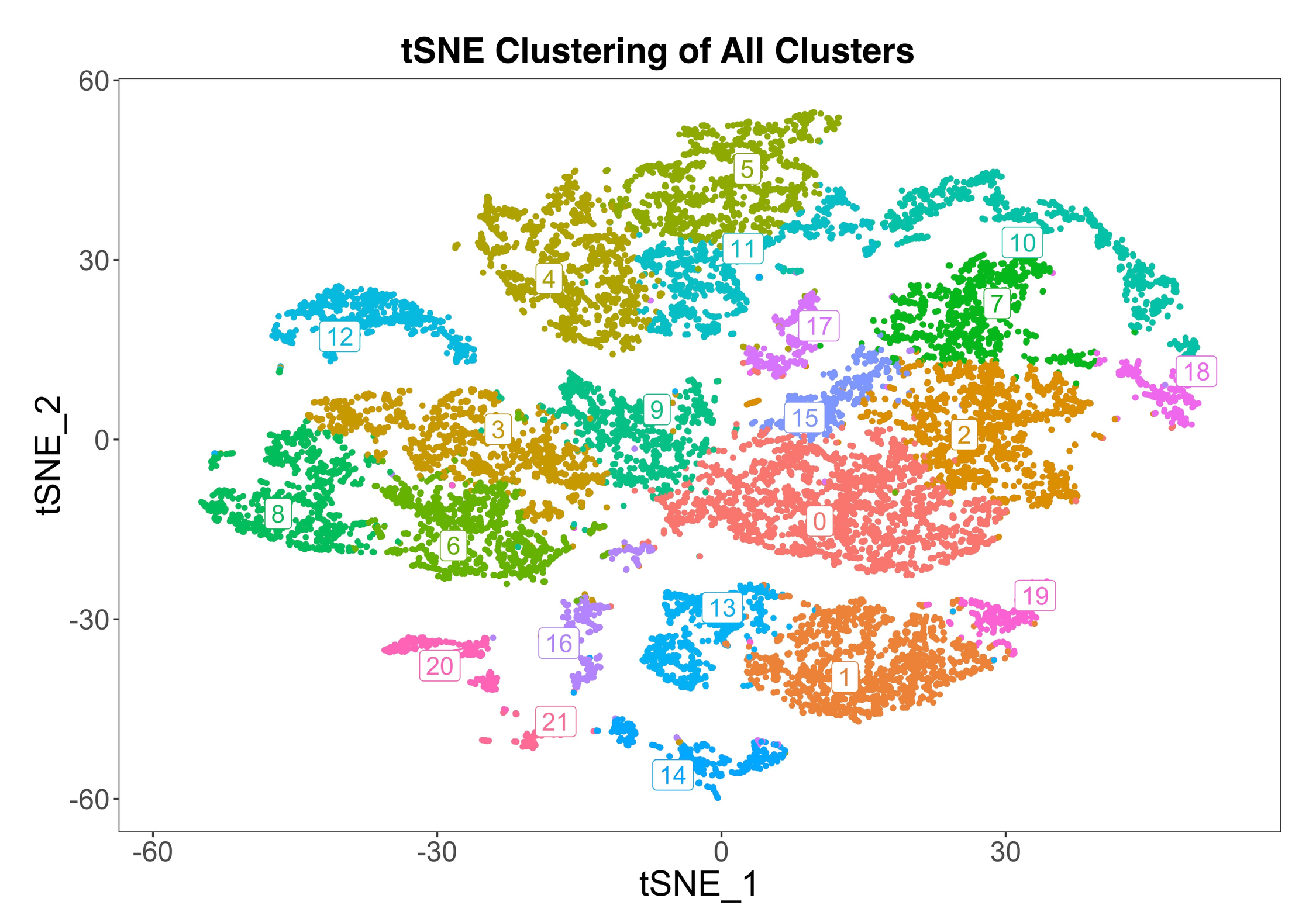

Supplement: S3 Fig — Representative clustering of all cells with >500 detected genes and >2000 UMIs, based on most significant principal components, color-coded by cell cluster. (TIF) [file pgen.1007810.s003.tif]

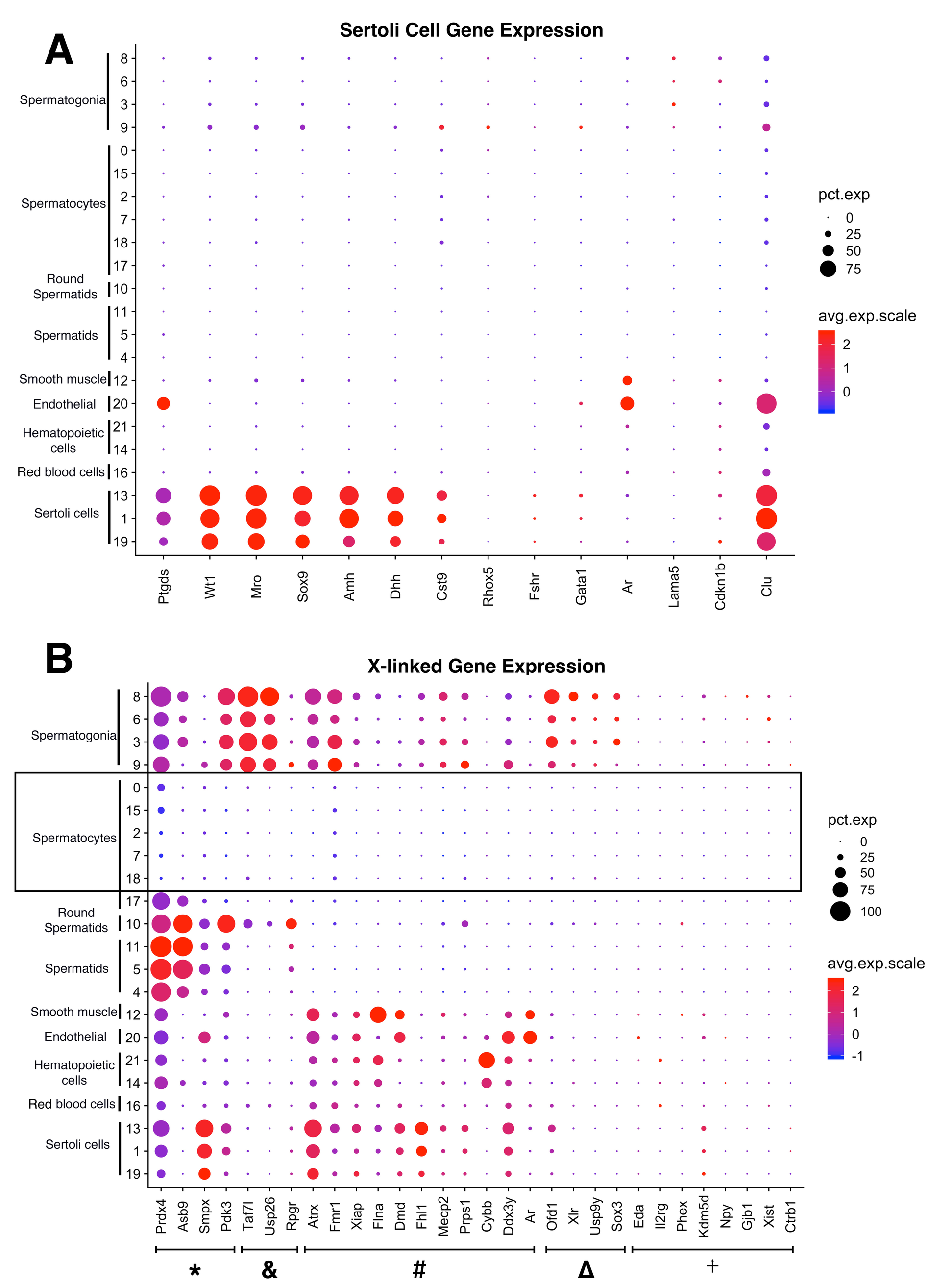

Supplement: S4 Fig — A) Dot plot representation of immature and mature Sertoli cell marker genes per cell cluster as determined in S3 Fig. Canonical immature Sertoli cell markers include Amh and Dhh, while mature markers include Gata1 and Clu (Spg-2). Wt1 and Sox9 are markers of all Sertoli cells [25–28]. Notably, for mature Sertoli cell marker genes which are only detected in a small percentage of cells in the cluster (as indicated by dot size), including Fshr and Gata1, expression in those cells is quite high as indicated by the dot’s red color. B) Dot plot representation of a sampling of X-linked genes per cell cluster as determined in S3 Fig. Four general patterns of expression are apparent: genes denoted by the asterisk (*) are detected robustly in spermatogonia, spermatids, and several types of somatic cells. Genes denoted by an ampersand (&) are detected robustly in spermatogonia and round spermatids. Genes denoted by the pound sign (#) are detected robustly in spermatogonia and several types of somatic cells. Genes denoted by the delta sign (Δ) are detected robustly in spermatogonia only. Genes denoted by a cross (⍏) are not robustly detected in any cell types. Overall, despite the different patterns of expression, spermatocytes have uniformly low detection of X-linked genes, as is expected due to meiotic sex chromosome inactivation. (TIF) [file pgen.1007810.s004.tif]

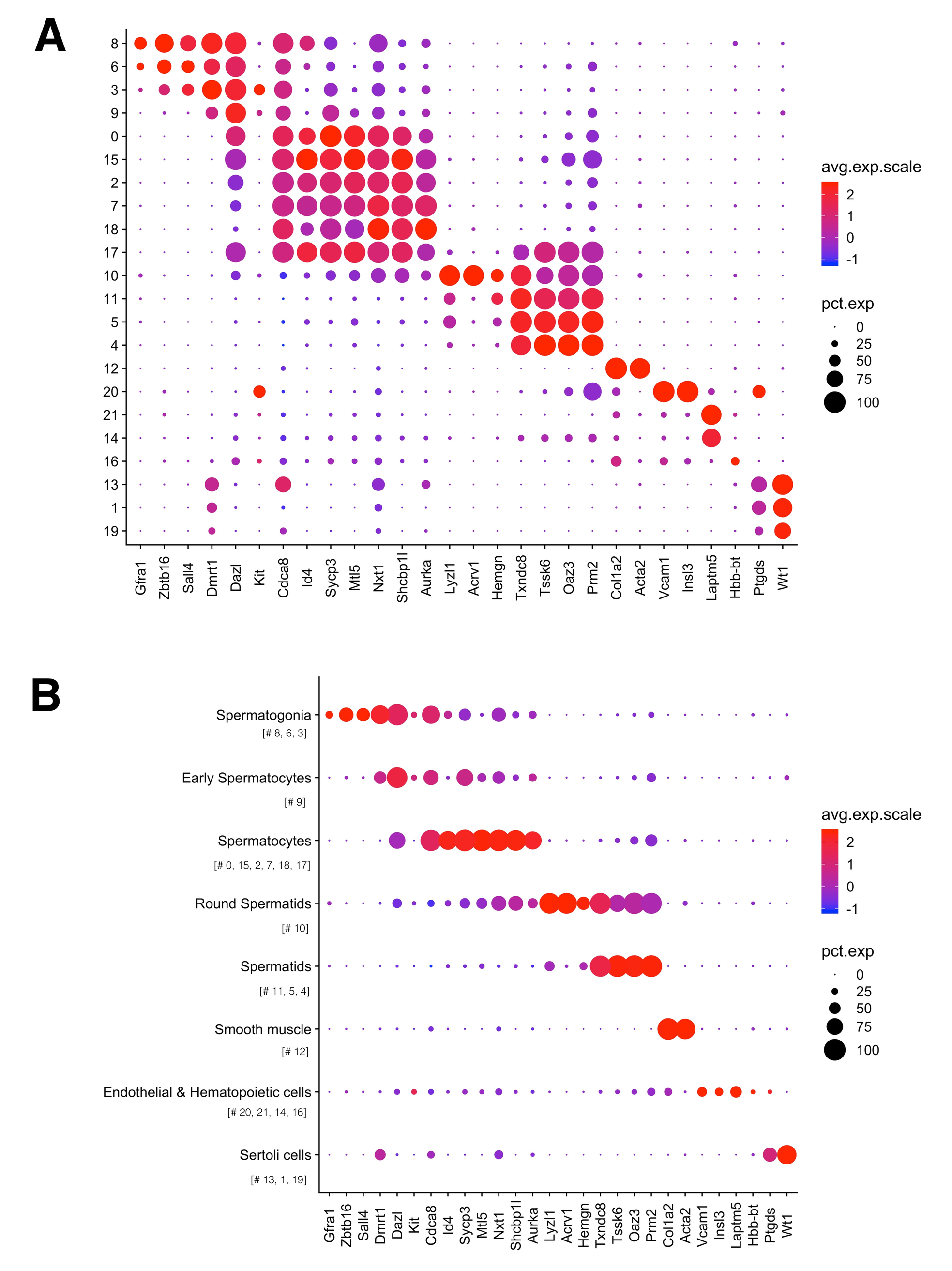

Supplement: S5 Fig — A) Dot plot representation of known marker genes per cell cluster determined in S3 Fig. B) Dot plot representation of known marker genes per cell type (supercluster) determined in Fig 2B. (TIF) [file pgen.1007810.s005.tif]

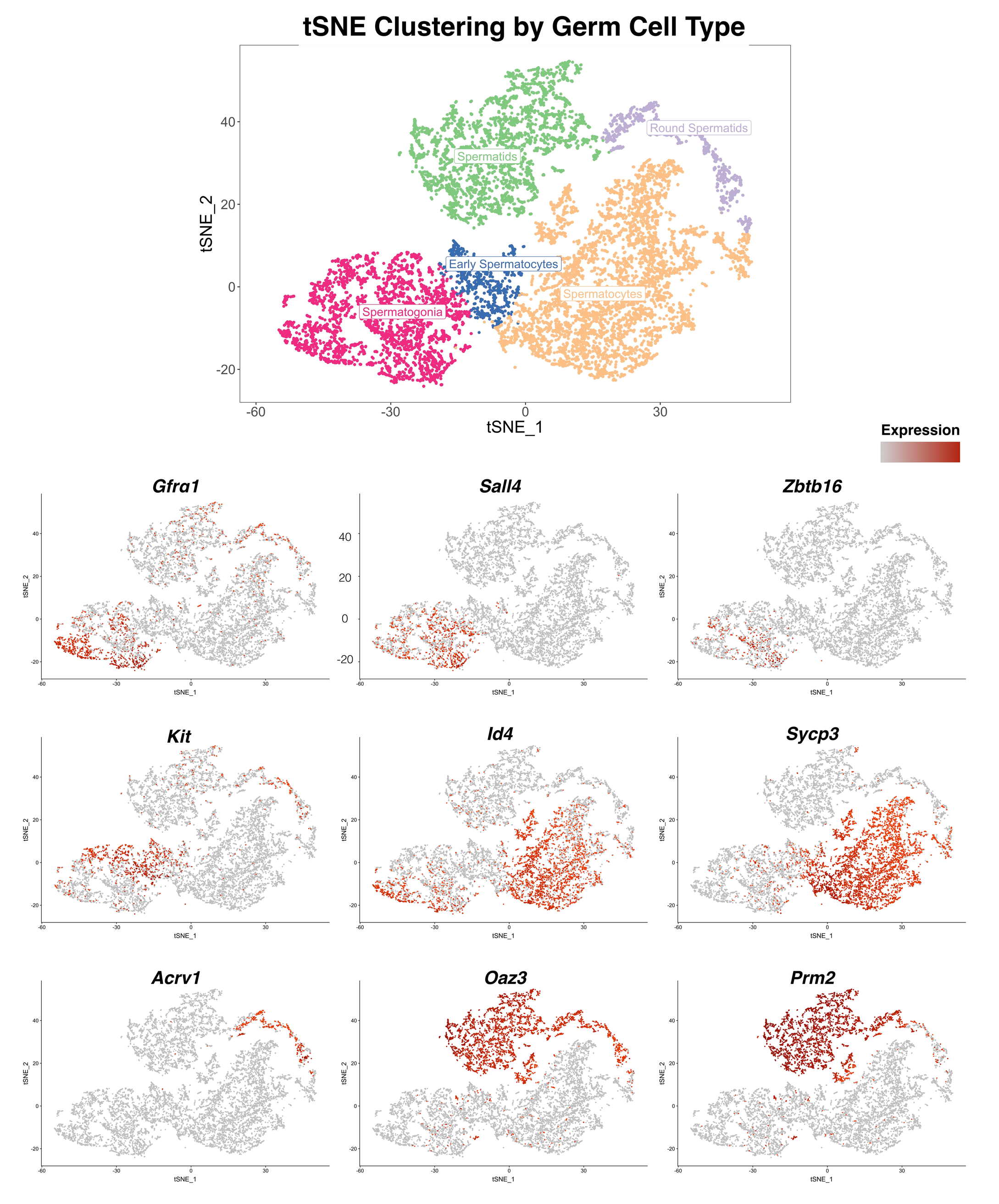

Supplement: S6 Fig — tSNE plot of germ cells from all libraries colored by cell type (top), as well as tSNE plots annotated with notable germ cell marker gene expression (bottom). (TIF) [file pgen.1007810.s006.tif]

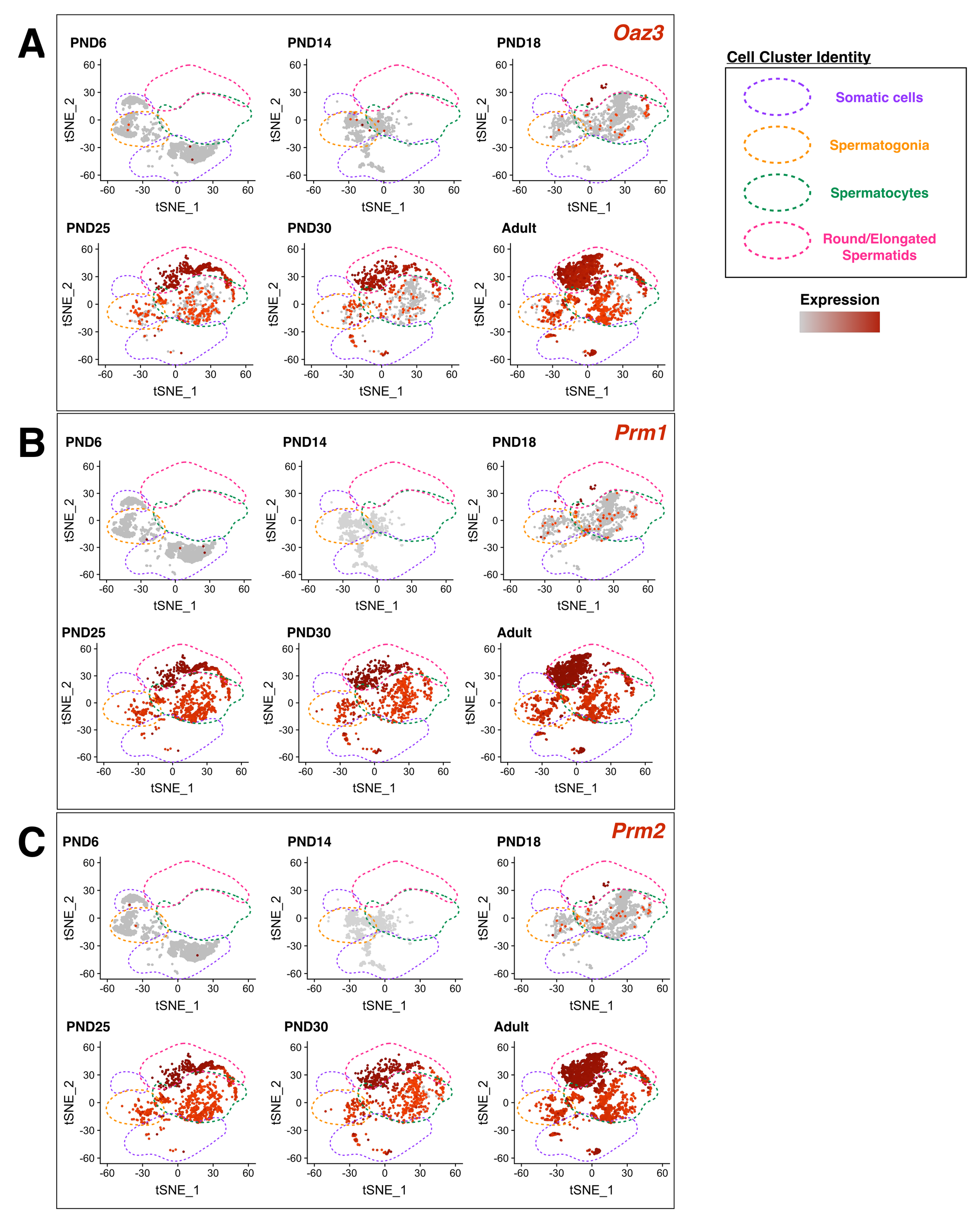

Supplement: S7 Fig — Representative tSNE plots of A) Oaz3, B) Prm1, and C) Prm2, showing detection of the indicated gene across all cell types in older samples only. Overlaid dotted lines indicate cell cluster identity by color. While minimal detection of example genes is observed in a small percentage of PND18 cells, robust expression does not become apparent until PND25, the first sample in which spermatids are present. Crucially, endogenous expression of these genes is not expected in somatic cell types, and detection of these transcripts in somatic cell types is therefore additional evidence of contaminating cell-free RNA. (TIF) [file pgen.1007810.s007.tif]

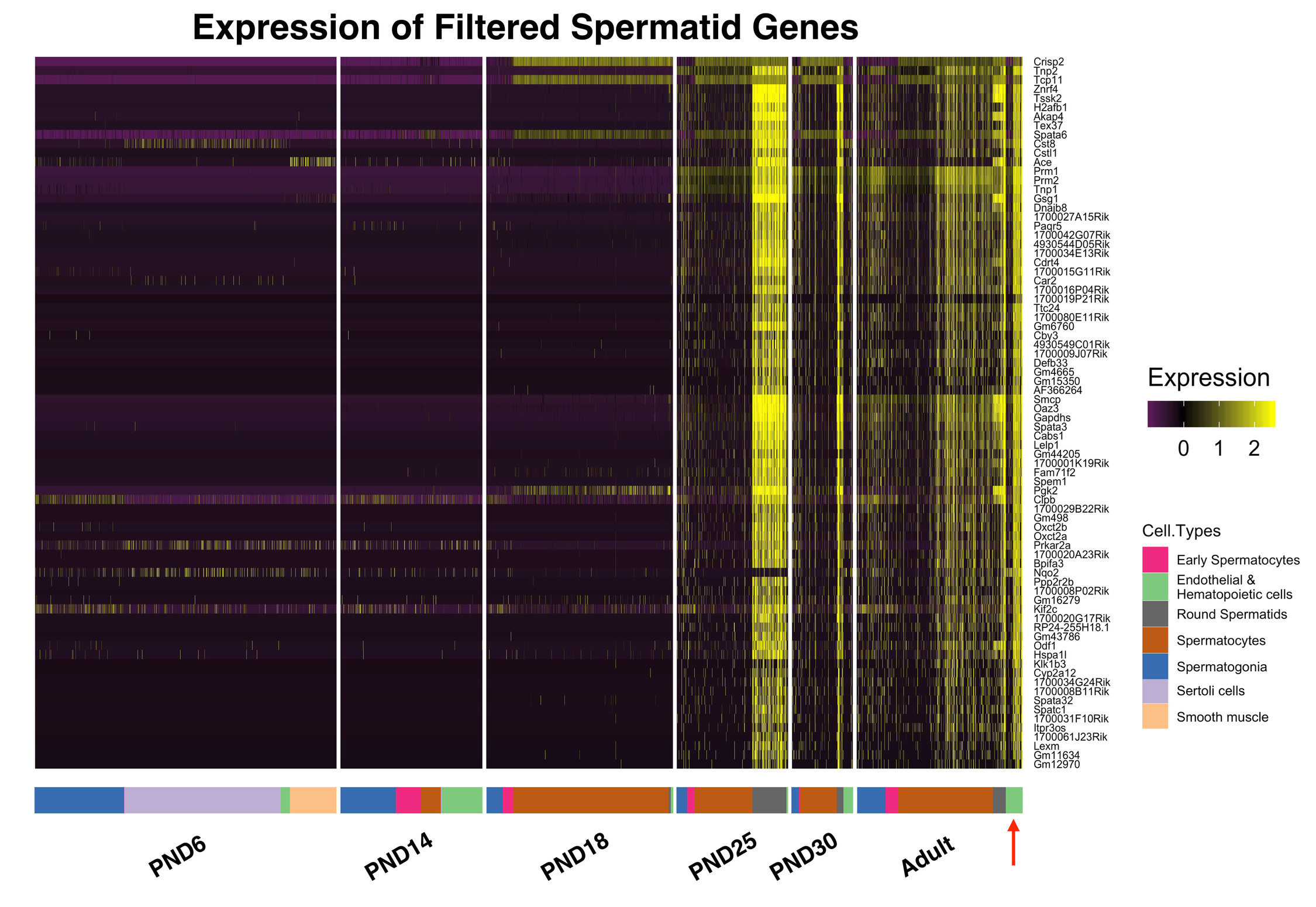

Supplement: S8 Fig — Heatmap of filtered spermatid genes per cell type (indicated by color bar), excluding elongated spermatids. Expression is scaled, ranging from 0 to 2, and excludes elongated spermatids. Contaminating spermatid gene detection is observed in somatic and germ cell types only in libraries in which spermatids are present (PND25 and later), suggesting that the detection of these genes is due to lysis of spermatids in the original material, and not premature expression of spermatid genes, which would have likely been observed in PND14 and PND18 as well. As is also observed in S7 Fig, endogenous expression of these genes is not expected in somatic cell types—denoted in this heatmap by a red arrow in the adult library—and is therefore additional evidence to support that detection of these transcripts in somatic cell types is due to contaminating cell-free RNA. (TIF) [file pgen.1007810.s008.tif]

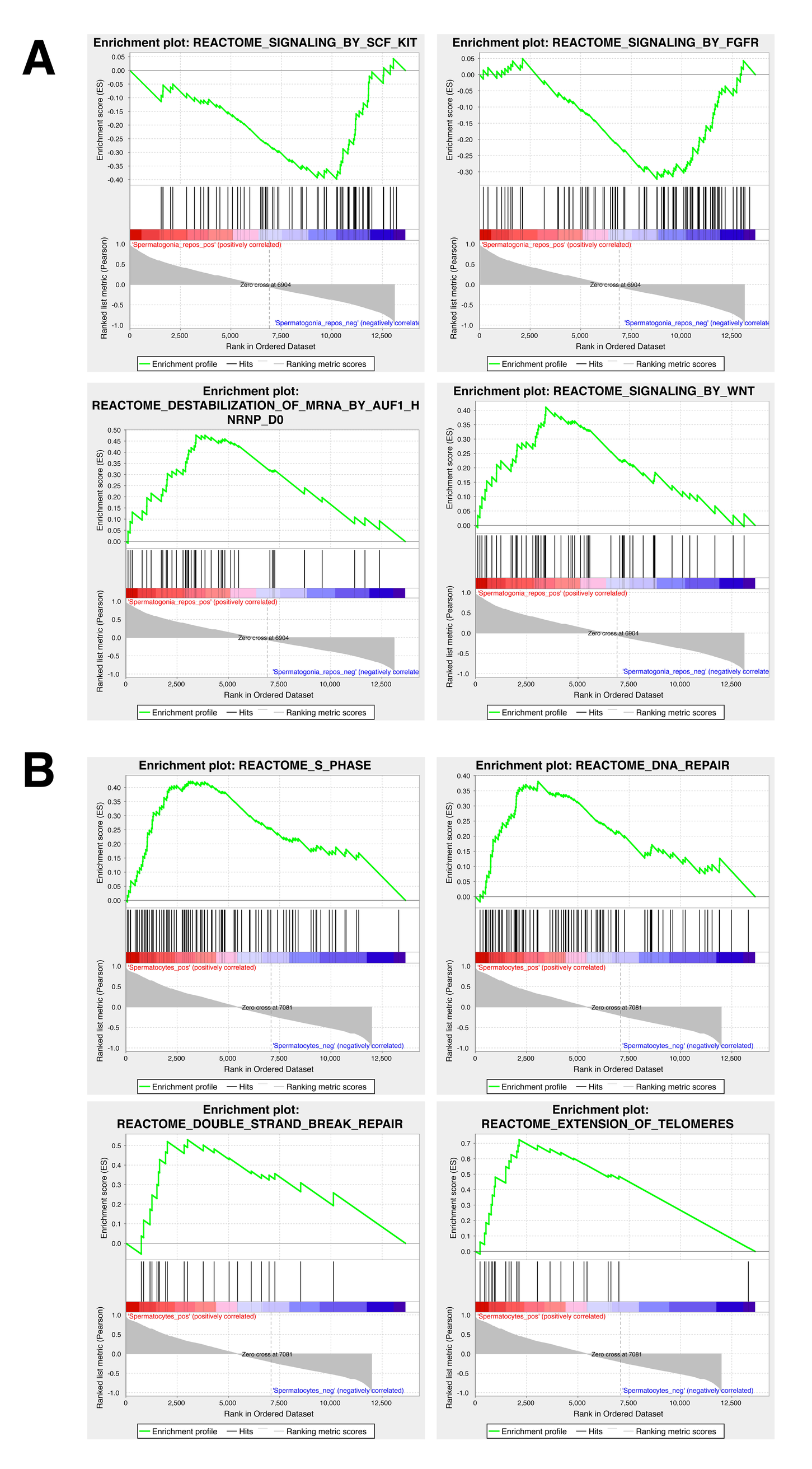

Supplement: S9 Fig — A) Enrichment plots for selected Reactome database pathways in spermatogonia. Pathways “SIGNALING_BY_SCF_KIT” and “SIGNALING_BY_FGFR” show negative correlation with developmental time, while pathways “DESTABILIZATION_OF_MRNA” and “SIGNALING_BY_WNT” show positive correlation with developmental time. B) Enrichment plots for selected Reactome database pathways in spermatogonia. All pathways shown demonstrate positive correlation with developmental time. (TIF) [file pgen.1007810.s009.tif]

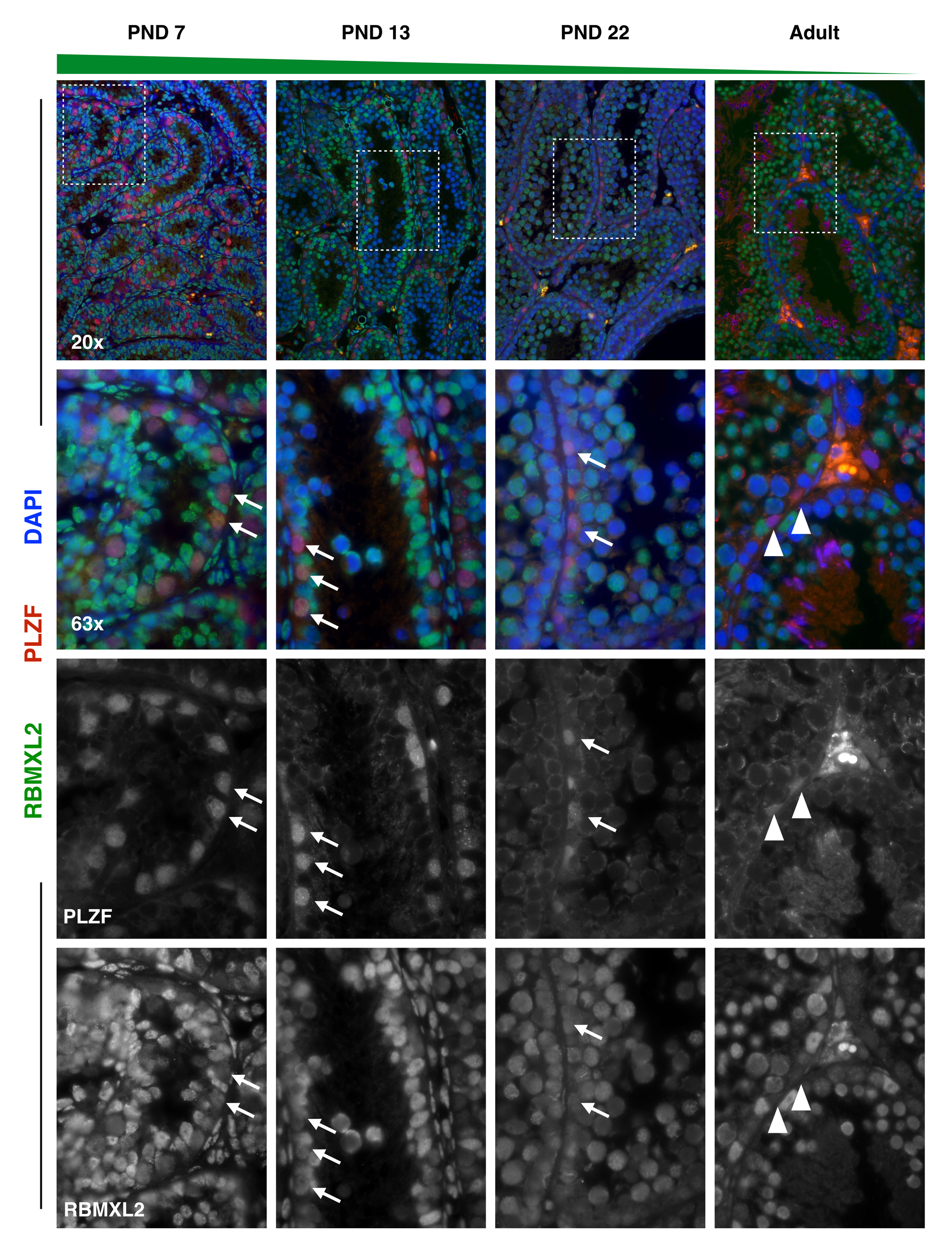

Supplement: S10 Fig — Spermatogonial marker PLZF (red) and RBMXl2 (green) were stained in 5μm testis tissue sections from mice ages PND7, PND13, PND22, and adult. DAPI (blue) denotes nuclei. RBMXL2 protein expression decreases in PLZF+ spermatogonia with age. High-RBMXL2-expressing spermatogonia are indicated by full arrows with a line, while low-RBMXL2-expressing cells are indicated by arrowheads. Individual channels are represented in gray scale. (TIF) [file pgen.1007810.s010.tif]
